# Atrial fibrillation and breast cancer—Vicious twins? A systematic review and meta-analysis

**DOI:** 10.3389/fcvm.2023.1113231

**Published:** 2023-03-10

**Authors:** Xiongda Yao, Qingwen Hu, Xiao Liu, Qing Ling, Yurong Leng, Huilei Zhao, Peng Yu, Jianyong Ma, Yujie Zhao, Menglu Liu, Renqiang Yang

**Affiliations:** ^1^Department of Cardiology, The Second Affiliated Hospital of Nanchang University, Nanchang, Jiangxi, China; ^2^Department of Cardiology, Sun Yat-Sen Memorial Hospital of Sun Yat-Sen University, Guangzhou, Guangdong, China; ^3^Guangdong Province Key Laboratory of Arrhythmia and Electrophysiology, Guangzhou, Guangdong, China; ^4^The Affiliated Stomatological Hospital of Nanchang University, Nanchang, Jiangxi, China; ^5^Department of Anesthesiology, The Third Hospital of Nanchang, Nanchang, Jiangxi, China; ^6^Department of Endocrine, The Second Affiliated Hospital of Nanchang University, Nanchang, Jiangxi, China; ^7^Department of Pharmacology and Systems Physiology, University of Cincinnati College of Medicine, Cincinnati, OH, United States; ^8^Department of Cardiology, Seventh People's Hospital of Zhengzhou, Zhengzhou, Henan, China

**Keywords:** atrial fibrillation, breast cancer, incidence, prevalence, association

## Abstract

**Background:**

Epidemiological studies suggest a bidirectional association between atrial fibrillation and breast cancer. This study aimed to conduct a meta-analysis to elucidate the prevalence of atrial fibrillation among breast cancer patients, and the bidirectional association between atrial fibrillation and breast cancer.

**Methods:**

PubMed, the Cochrane Library, and Embase were searched to identify studies reporting the prevalence, incidence, and bidirectional association between atrial fibrillation and breast cancer. The study was registered with PROSPERO (CRD42022313251). Levels of evidence and recommendations were assessed by the Grading of Recommendations Assessment, Development and Evaluation (GRADE).

**Results:**

Twenty-three studies (17 retrospective cohort studies, 5 case-control studies and 1 cross-sectional study) involving 8,537,551 participants were included. Among patients with breast cancer, the prevalence of atrial fibrillation was 3% (11 studies; 95% CI: 0.6 to 7.1%) and the incidence was 2.7% (6 studies; 95% CI: 1.1 to 4.9%). Breast cancer was associated with increased risk of atrial fibrillation (5 studies; hazard ratio [HR]: 1.43, 95% CI: 1.12 to 1.82, I^2^ = 98%). Atrial fibrillation was also significantly associated elevated risk of breast cancer (5 studies HR: 1.18, 95% CI: 1.14 to 1.22, I^2^ = 0%). Grade assessment shown low certainty of the evidence for the risk of atrial fibrillation and moderate certainty of the evidence for the risk of breast cancer.

**Conclusion:**

Atrial fibrillation is not uncommon in patients with breast cancer and vice versa. There is a bidirectional association between atrial fibrillation (low certainty) and breast cancer (moderate certainty).

## Graphical Abstract

Cartoon depicting the association between atrial fibrillation and breast cancer. 
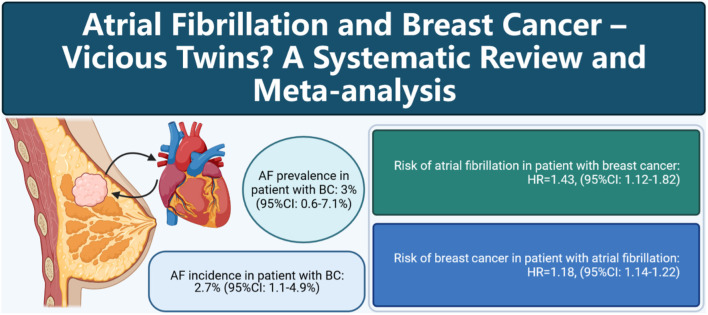

## Introduction

Breast cancer is the most frequent cancer amongst women worldwide, accounting for about 24.5 percentage of all cancers (1). Thanks to advances in treatment, the 5-year survival rate for breast cancer patients has significantly increased to 89% (2). However, cardiovascular disease is increasingly becoming a barrier to optimal outcomes for breast cancer patients (3).

Atrial fibrillation, the most common arrhythmia, is an important cause of embolism and death from cardiovascular diseases, with a global prevalence of approximately 2–4%. Age is a strong risk factor for the incidence of atrial fibrillation. The prevalence of atrial fibrillation tends to increase with the increase in human life expectancy (4, 5). One study showed that malignant neoplasms (23.1%) were the most common cause of death in patients with atrial fibrillation (6).

Recently, many epidemiological studies have shown an increased risk of new-onset atrial fibrillation in women with breast cancer (7–15), while other studies have shown an increased risk of breast cancer in women with atrial fibrillation (13, 16–20). It is important to note that these studies have not yielded consistent results. The incidence and prevalence of atrial fibrillation in breast cancer are unknown, and it is unclear whether there is a bidirectional association between atrial fibrillation and breast cancer. Thus, we conducted a systematic review and meta-analysis to elucidate the prevalence and incidence of atrial fibrillation among breast cancer patients and the association between atrial fibrillation and breast cancer.

## Methods

### Protocol registration

The study was registered in the PROSPERO (International prospective register of systematic reviews. https://www.crd.york.ac.uk/PROSPERO/ -registration number- CRD42022313251). This meta-analysis was performed according to the PRISMA 2021 guideline for systematic review and meta-analysis (21) (Supplementary Table S1).

### Search strategy

We systematically searched the PubMed, EMBASE, Cochrane Library online database up to February 25, 2022 with no language restriction. The gray literatures, published in American College of Cardiology (ACC)/American Heart Association (AHA), European Society of Cardiology (ESC) were also reviewed. The search MeSH items and keywords were as follows: (“Breast Neoplasms” [Mesh] OR “breast cancer” OR “breast cancers” OR “breast tumors” OR “breast tumors” OR “mammary Cancer” OR “mammary cancers” OR “mammary gland cancers” OR “mammary gland cancer”) AND (“Atrial Fibrillation” [Mesh] OR “atrial fibrillations” OR “auricular fibrillation”) OR (“Mortality” [Mesh] OR “Mortalities” OR “Case Fatality Rate” OR “Case Fatality Rates” OR “Death Rate” OR “Death Rates”). The detailed search strategy is shown in Supplementary Table S2.

### Selection criteria

Eligible studies were as follows: (1) clinical trials or observational studies (cohort, case–control, or case-cohort or nest-case control) reported the prevalence or the incidence of atrial fibrillation in patients with breast cancer. (2) studies assessed the association between atrial fibrillation and breast cancer expressing with Relative Risk (RR)/HR and corresponding 95% CI or that can be calculated. For the incidence of atrial fibrillation or cancer, we only included cohort studies or the case cohort designed data. (3) studies assessed the association between atrial fibrillation and mortality in patients with breast cancers. Two authors (X-L and X.D-Y) independently reviewed the articles for eligibility. Any discrepancies were discussed until consensus.

Accordingly, studies that met the following criteria were excluded: (1) Studies that specifically looked at the influence of breast cancer-related treatments (e.g., radiotherapy chemotherapy and adjuvant therapy) on atrial fibrillation, as anticancer treatment was linked to atrial fibrillation incident by the previous studies (22–31). (2) Reviews, meta-analyses, practice guidelines, patents, cases, replies, comments, or editorial; (3) after contacting the author, the data needed for the article still cannot be obtained.

### Data extraction and quality assessment

The information was extracted from each study, including first author, publication year, country, mean age, study design, data source, follow-up, prevalence or incidence of atrial fibrillation or breast cancer, atrial fibrillation diagnosis, breast cancer diagnosis, sample size, any subgroup, effect size, and adjustments. Three author (X.D-Y and X-L Z.Q-T) extracted data. For studies that reported the prevalence of atrial fibrillation or breast cancer, the Joanna Briggs Institute (JBI) critical appraisal checklist was used to assess the study quality. The Newcastle-Ottawa quality scale (NOS) was used to quantify the quality of studies reporting association between breast cancer and atrial fibrillation. A score of JBI > 16 or NOS > 6 was regarded as acceptable quality.

### Statistical analysis

We used a random-effect model to summarize the estimates and 95% confidence intervals (CIs). For incidence of atrial fibrillation or breast cancer, the exact binomial (Clopper–Pearson) method was used to calculate 95%CI (32). Estimates were standardized using the Freeman–Tukey double arcsine transformation (33). For the effect of atrial fibrillation on mortality in breast cancer patients, estimates from each study were consolidated by the method of DerSimonian and Laird (34), which designated the weight of each study based on its variance. If a study reported results separately for subgroups (e.g., age), we combined the subgroup-specific estimates using a random-effect so that each study was represented only once in the forest plot. If one study reported one more effect size, we used the most informative one. In the association outcomes, considering the difference in study design, the evidence of cohort and case-control studies was pooled separately. In cohort studies, we treat standardized incidence ratios (SIRs) as equivalent to RR.

Heterogeneity was evaluated using the Higgins I-squared (I^2^) statistic (25, 50, and 75% represent low, moderate, and high heterogeneity, respectively) (35). Publication bias was addressed by Funel plot, and Egger's and Begg's test, with the results considered to be significant when *P* < 0.10 (36). For the purpose of appraising the robustness and reliability of the primary study outcomes, we also carried out sensitivity analyses by omitting each study in turn. Subgroup analysis was defined as mean age (< 65 vs. ≥65 years), region (America, Europe, and Asia), sample size (< 5,000 vs. ≥5,000), case (< 50 vs. ≥50) if enough studies were available (N > 10) considering the statistical power according to the guidelines. For the association of atrial fibrillation with breast cancer, an additional subgroup was stratified by hormone therapy. The statistical analysis was performed by the RevMan software, version 5.4.1 (The Cochrane Collaboration, Nordic Cochrane Center Copenhagen, Denmark) and Stata software, Version 16.0 (Stata Corp LP, College Station, Texas, USA). *P* < 0.05 double-sided was considered statistically significant. We evaluated the quality of evidence for each outcome using the GRADE method (37, 38). Two authors separately assessed the quality of evidence for each outcome. We used GRADEpro GDT to provide evidence profile tables. We present the results of the outcomes as described in the outcome metric type section, footnotes are used to indicate reasons for degradation or escalation of the quality of evidence.

## Results

### Literature search

**Figure 1:**
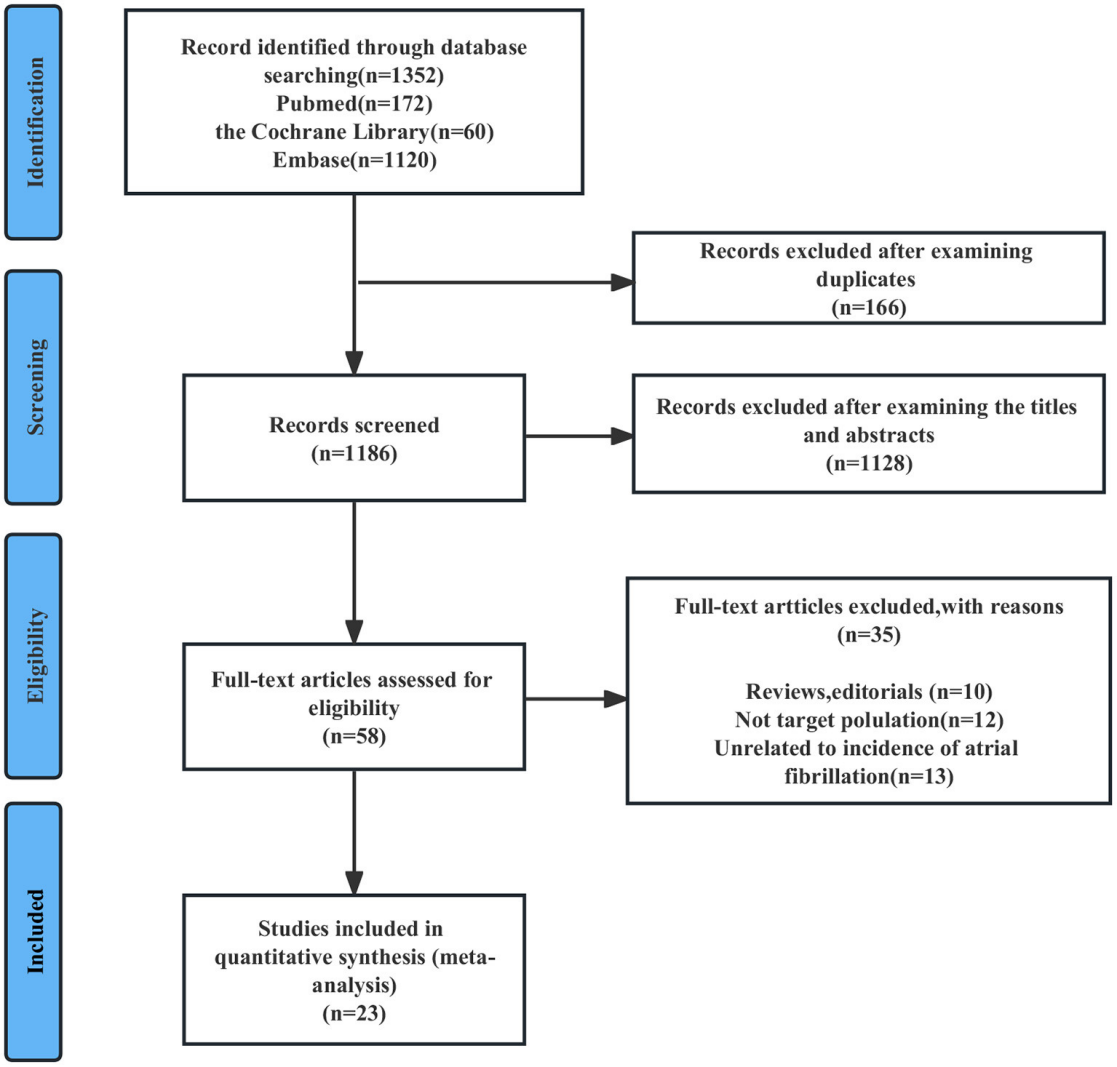
Flowchart of study selection for the bidirectional association between AF and BC. AF, atrial fibrillation; BC, breast cancer.

As shown in Figure 1, an initial online database search resulted in 1,352 abstracts. After excluding 166 duplicated records and 1,128 animal studies, *in vitro* studies or irrelevant studies based on the screening of titles/abstracts, 58 reports remained for full-text review. Thirty-six full-length studies were excluded for the following reasons: (1) reviews or editorials (*n* = 10); (2) did not include the target population (*n* = 12); or (3) did not include the target outcomes (*n* = 13). The excluded studies are listed in Supplementary Table S3. Finally, 23 studies were included in this meta-analysis.

### Study characteristics and quality

Table 1 summarizes the main characteristics of the included studies. Seventeen were retrospective cohort studies (7, 9–11, 13, 14, 31, 39, 40, 44–46), 5 were case-control studies (8, 12, 15, 41, 43), and 1 was a cross-sectional study (42). The follow-up time ranged from 0.24 to 19.1 years. Seventeen studies reported the prevalence or incidence of atrial fibrillation (7–12, 14, 15, 39–47), 5 studies reported an association between atrial fibrillation and breast cancer (16–20), 5 studies reported an association between breast cancer and atrial fibrillation (7, 10, 11, 13, 14) and 2 studies reported an association between atrial fibrillation and all-cause mortality in patients with breast cancer (11, 41). Overall, these studies were published between 2008 and 2021, the sample sizes ranged from 86 to 1,122,100 with a total of 8,537,551 individuals, nine studies were from North America (the US and Canada) (7, 8, 11, 15, 16, 20, 39–41), 7 studies were from Europe (9, 10, 12, 18, 19, 44, 46), and 7 studies were from Asia (13, 14, 17, 42, 43, 45, 47). The mean age varied from 51.9 to 78 years. Sixteen studies defined atrial fibrillation according to the International Classification of Diseases criteria (7, 10, 11, 13–15, 17–19, 39–41, 43, 44, 46, 47), and 7 studies were based on electrocardiogram reports (8, 9, 12, 16, 20, 42, 45). All studies defined breast cancer from medical records according to the International Classification of Diseases (7, 8, 10, 11, 31, 39–43, 45–47). Among the studies reporting atrial fibrillation and breast cancer, four studies reported crude risk estimates (9, 12, 17, 18), and the other adjustments varied considerably. Ten studies adjusted for significant confounding factors (7, 8, 10, 11, 13–16, 19, 20), including age, sex, smoking, alcohol consumption, hypertension, diabetes, and heart failure, and 5 studies adjusted for confounders associated with anticancer therapy, including radiation therapy, chemotherapy, surgery, therapy after lymph node surgery, and hormone replacement therapy (11, 16, 19, 20, 41).

**Table 1:** Characteristics of included studies in this meta-analysis.

**References, country**	**Sample of size, N**	**AF diagnosis**	**BC diagnosis**	**Study design follow up (year)**	**Mean age (years)**	**AF cases, *N***	**New-onset AF case, *N***	**Outcomes reported**	**Adjustments**
**Epidemiology**									
Abdel-Qadir et al. (7, 39), Canada	90104	ICD	ICD	Retrospective cohort 10	61	1,713	NR	Prevalence of AF	
Apte et al. (8), USA	1872	ECG	ICD	Case-control	NR	54	NR	Prevalence of AF	Age, hypertension, diabetes, BMI, and male sex
Boerman et al. (9), Netherland	350	ECG	ICD	Retrospective cohort 10	63	NR	11	Incidence of AF	
Ezaz et al. (40), USA	1664	ICD	ICD	Retrospective cohort 3	73.6	90	NR	Prevalence of AF	
Han et al. (41), USA	305300	ICD	ICD	Case-control	NR	10,741	NR	Prevalence of AF mortality	Age, race, gender, income, insurance type, year, hospital region, hospital type, hospital bed size, primary tumor site, multiple metastatic sites (≥2), major operating room procedure, chemotherapy, long- term anticoagulants, metastatic sites, coronary artery disease, prior stroke, acquired immune deficiency syndrome (AIDS), alcohol abuse, deficiency anemias, rheumatoid arthritis, chronic blood loss anemia, congestive heart failure, chronic pulmonary disease, coagulopathy, depression, uncomplicated diabetes, diabetes with chronic complications, drug abuse, hypertension, hypothyroidism, obesity, lymphoma, fluid and electrolyte disorders, other neurological disorders, paralysis, peripheral vascular disorders, psychoses, pulmonary circulation disorders, renal failure, ulcer disease, valvular disease, weight loss and liver disease
Li et al. (42), China	5677	ECG	ICD	Cross-sectional	NR	93	NR	Prevalence of AF	
Lin et al. (43), China Taiwan	1188	ICD	ICD	Case-control	75.8	40	NR	Prevalence of AF	
Mery et al. (44), France	682	ICD	ICD	Retrospective cohort 5	62.5	NR	8	Incidence of AF	
Guzzetti et al. (12), Italy	502	ECG	ICD	Case-control	60.5	10	NR	Prevalence of AF	Ps for age
Okura et al. (45), Japan	2699	ECG	ICD	Retrospective cohort 10	NR	20	NR	Prevalence of AF	
Ording et al. (46), Denmark	62376	ICD	ICD	Retrospective cohort 5	62.3	567	NR	Prevalence of AF	
Yamashita et al. (47), Japan	86	ICD	ICD	Retrospective cohort	58.8	2	NR	Prevalence of AF	
Zubair et al. (15), USA	7734604	ICD	ICD	Case-control	NR	1,122,100	NR	Prevalence of AF	Age, DM, hypertension, CAD, obesity, congestive heart failure valve disorder, thyrotoxicosis, hypothyroidism, collagen vascular disease, and chronic pulmonary disease
**BC to AF**									
Abdel-Qadir et al. (7, 39), Canada	68113	ICD	Pathology ICD	Retrospective cohort 10	60	NR	5,040	Incidence of AF risk of AF	Age, year of cohort entry, rural residence, ischemic heart disease, heart failure, diabetes, hypertension, peripheral vascular disease, stroke, chronic obstructive pulmonary disease, and chronic kidney disease, number of family physician, specialist claims (updated annually)
D'Souza et al. (10), Denmark	74155	ICD	ICD	Retrospective cohort 3	62	NR	987	Incidence of AF risk of AF	Hypertension, ischemic heart disease, heart failure, diabetes mellitus, thyroid disease, chronic kidney failure, peripheral arterial disease, chronic obstructive pulmonary disease, chronic liver disease
Guha et al. (11), USA	85423	ICD	ICD	Retrospective cohort 1	78	NR	2,993	Incidence of AF risk of AF mortality	Age, race, Hispanic, registry, urban, poverty, laterality, grade, AJCC stage, surgical, lymph node surgery therapy, radiation therapy, breast tumor subtype based on combination receptor status, hypertension, Diabetes, obesity, history of ischemic stroke/transient ischemic attack, hyperlipidemia, history of congestive heart failure, history of myocardial infarction, history of lung disease, smoking, history of depression, history of anemia, anthracycline vs. not, Her2inhib vs. not, cyclophosphamide vs not, taxanes vs. not, platinum compounds vs. not, hormonal therapy vs. not (only who have part D), beta-blockers, angiotensin-converting enzyme (ACE) inhibitors/angiotensin II receptor blockers (ARBs), spironolactone/eplerenone vs. not
Saliba et al. (13), Israel^*^	11220	ICD	ICD	Retrospective cohort 0.24	NR	NR	NR	Risk of AF risk of BC	Age, sex, smoking, alcohol consumption, physical activity, education, medications use (aspirin, statins, and anticoagulants), and comorbidities (hypertension, diabetes, congestive heart failure, and cardiovascular disease)
Yun et al. (14), Korea	81066	ICD	ICD	Retrospective cohort 4.5	51.9	NR	1,212	Incidence of AF risk of AF	Age, sex, smoking, drinking, regular exercise, socioeconomic status, DM, hypertension, dyslipidemia, BMI, and CKD
**AF to BC**									
Conen et al. (16), USA	NR	ECG	ICD	Retrospective cohort 19.1	NR	NR	NR	Risk of BC	Age, randomized treatment assignment, educational level, race/ethnicity, and height at study entry and time-dependent measures for BMI, hypertension, hypercholesterolemia, DM, smoking status, number of cigarettes smoked per day, alcohol consumption, physical activity, hormone replacement therapy use, presence of a recent cancer screening test, and incident non-fatal cardiovascular events (CHF, MI, or stroke).
Hung et al. (17), China Taiwan	NR	ICD	ICD	Retrospective cohort 3.1	NR	NR	NR	Risk of BC	
Ostenfeld et al. (18), Denmark	NR	ICD	ICD	Retrospective cohort 3.4	NR	NR	NR	Risk of BC	
Vinter et al. (19), Denmark	NR	ICD	ICD	Retrospective cohort 16.7	NR	NR	NR	Risk of BC	Age, BMI, cumulative alcohol consumption, smoking duration, tobacco consumption, hypertension, hypercholesterolemia, DM, physical activity, hormone treatment (women only), educational level, education length, smoking status, and Healthy Nordic Food Index
Wassertheil-Smoller et al. (20), USA	4,376	ECG self-reported	ICD	Retrospective cohort 15.3	NR	NR	NR	Risk of BC	Age, race, educational level, income, marital status, physical activity level, parity, age at menopause, hormone therapy use, hysterectomy, diabetes, and history of cardiovascular disease (MI, stroke, transient ischemic attack, angina, or revascularization)

AF, atrial fibrillation; BC, breast cancer; BMI, body mass index; CAD, coronary artery disease; CHF, chronic heart failure; CKD, chronic kidney disease; DM, diabetes mellitus; ECG, electrocardiogram; HR, hazard ratio; ICD, International Classification of Diseases; ICPC, International Classification of Primary Care; MI, myocardial infarction; NR, not reported; OR, odds ratio;

* “BC to AF” and “AF to BC”.

According to the JBI critical appraisal checklist, 17 studies (7–12, 14, 15, 39–47) that reported the prevalence of atrial fibrillation met at least six of the nine criteria, indicating that the articles used a rigorous approach (Supplementary Table S4). Based on the NOS, all 13 studies (7–11, 13–20) involving the association of atrial fibrillation and breast cancer were considered moderate to high quality, with a score range of 6–9, and one study in which the control group included hospital-based non-tumor patients rather than community-derived controls and adjusted for confounders had a score of 5 (12) (Supplementary Table S5).

### Atrial fibrillation and breast cancer

#### Epidemiology of atrial fibrillation in patients with breast cancer

**Figure 2:**
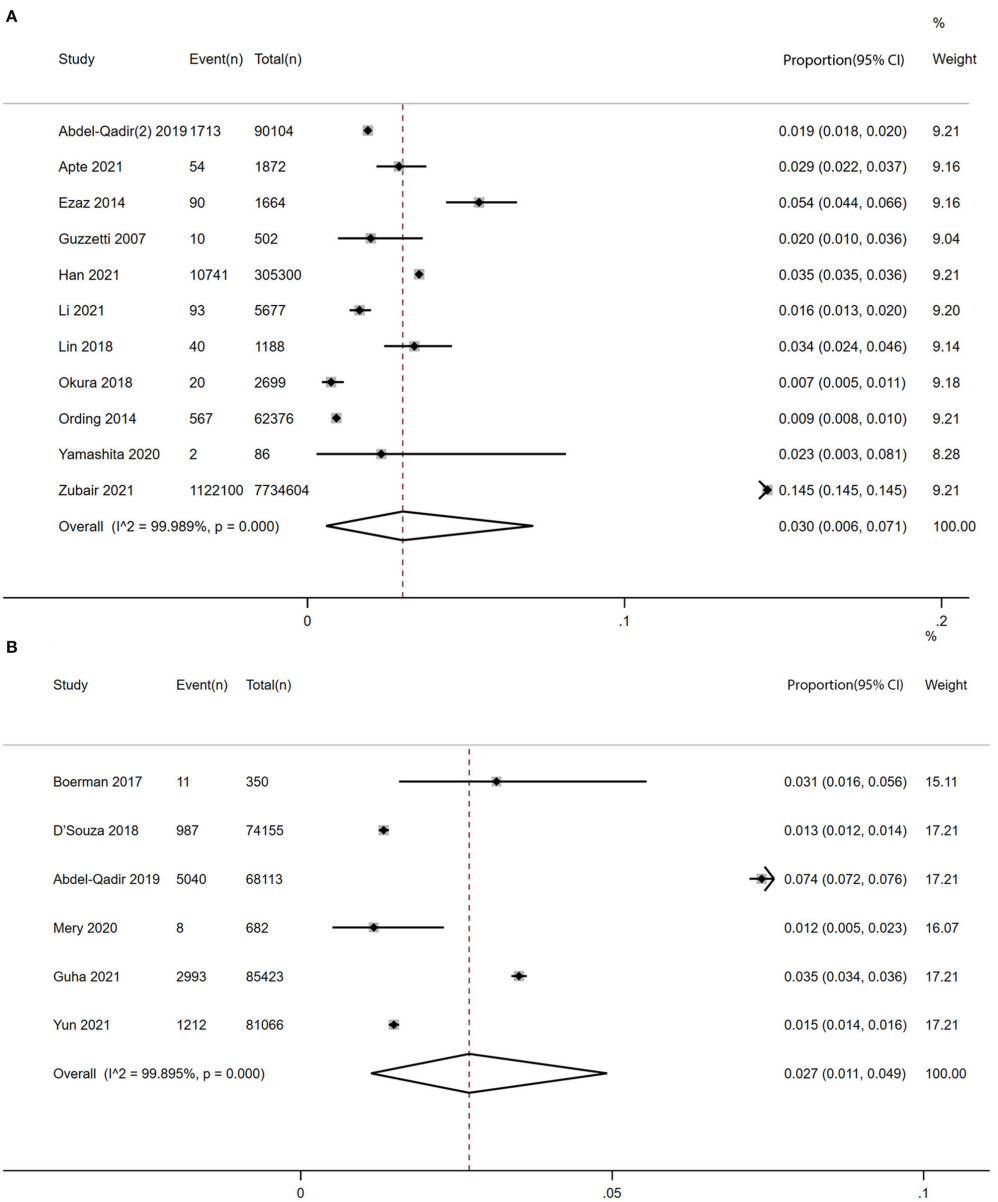
Forest plot of prevalence and incidence of AF in patients with BC [**(A)** prevalence vs. **(B)** incidence]. Inside the forest plot, the rhombus indicates the pooled estimate. Black squares and black vertical lines indicate 95%CIs around the effect size estimate. AF, atrial fibrillation; BC, breast cancer.

Ten studies (8, 12, 15, 39–42, 45–47) with 8,204,884 participants reported the prevalence of atrial fibrillation among patients with breast cancer. The pooled prevalence of atrial fibrillation was 3% (95% CI: 0.6 to 7.1%), with high heterogeneity (I^2^ = 99.99%) (Figure 2A).

**Figure 3:**
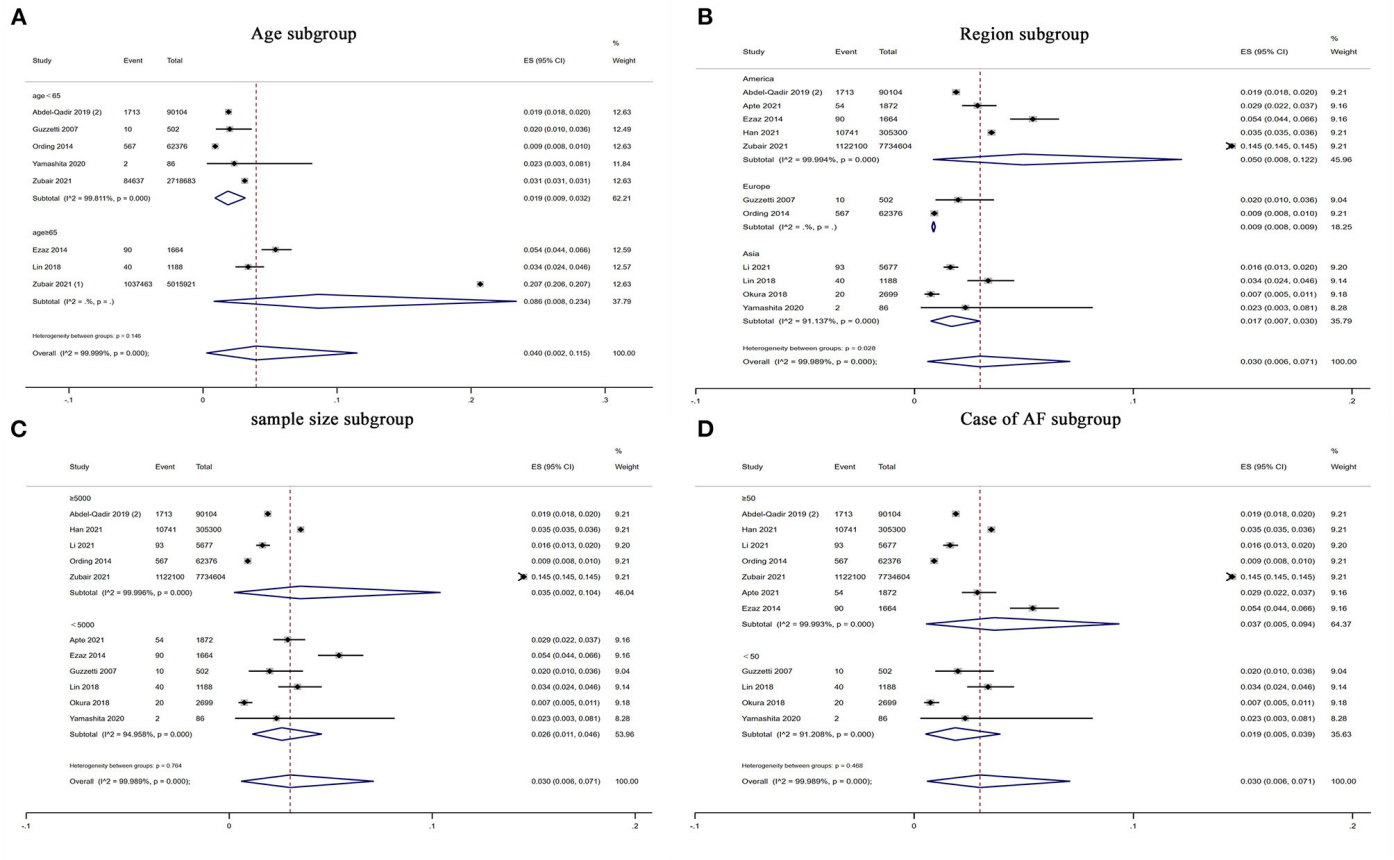
Forest plot of subgroup analysis for prevalence of AF in patients with BC. There were **(A)** age subgroup, **(B)** region subgroup, **(C)** sample size subgroup and **(D)** case of AF subgroup. Inside the forest plot, the rhombus indicates the pooled estimate. Black squares and black vertical lines indicate 95%CIs around the effect size estimate. AF, atrial fibrillation; BC, breast cancer.

In the subgroup analysis, the prevalence of atrial fibrillation (3%) was highest among breast cancer patients in the Americas (5%), followed by Asia (1.7%), and Europe (0.9%) (*P* for subgroup difference = 0.028) (Figure 3B). Older (age ≥ 65) patients with breast cancer (8.6%) showed a higher prevalence of atrial fibrillation than younger (age < 65) patients with breast cancer (1.9%) (Figure 3A). Moreover, the prevalence of atrial fibrillation was higher among breast cancer patients (3.5%) in studies with larger sample sizes (≥5,000) than among breast cancer patients (2.6%) in studies with smaller sample sizes (< 5,000) (Figure 3C). The prevalence of atrial fibrillation was higher among breast cancer patients (3.7%) in studies with a greater number of atrial fibrillation cases (≥50) than among breast cancer patients (1.9%) in studies with a smaller number of atrial fibrillation cases (< 50) (Figure 3D). However, there were no significant differences regarding age (*P* = 0.15), sample size (*P* = 0.76) or number of atrial fibrillation cases (*P* = 0.47) (Table 2).

**Table 2:** Subgroup analysis of prevalence of atrial fibrillation in patient with breast cancer.

**Items**		**Number of studies**	**Proportion (95%CI)**		***P***	P^*^h (%)	***P* ^#^**
Result of primary analysis		11	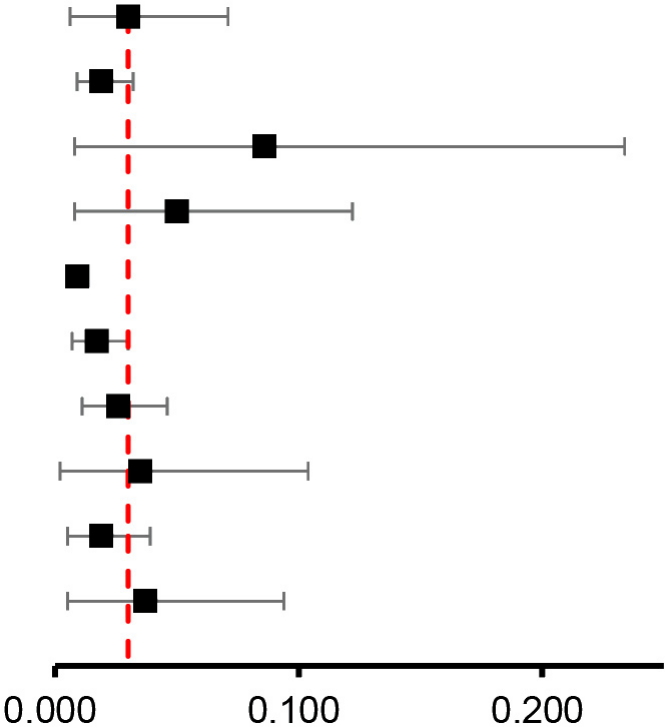	0.030 (0.006–0.071)			
Mean age	< 65 years	5		0.019 (0.009–0.032)	< 0.001	99.8	0.15
	≥65 years	3		0.086 (0.008–0.234)	0.007	-	-
Region	America	5		0.050 (0.008–0.122)	< 0.001	99.9	0.028
	Europe	2		0.009 (0.008–0.009)	-	-	-
	Asia	4		0.017 (0.007–0.030)	< 0.001	91.1	-
Sample size	< 5,000	6		0.026 (0.011–0.046)	< 0.001	95	0.76
	≥5,000	5		0.035 (0.002–0.104)	< 0.001	99.9	-
Cases	< 50	4		0.019 (0.005–0.039)	< 0.001	91.2	0.47
	≥50	7		0.037 (0.005–0.094)	0.002	99.9	-

* *P* for within-group heterogeneity,

# *P* for subgroup difference.

Six studies (7, 9–11, 14, 44) with a total of 309,789 participants reported the incidence of atrial fibrillation among patients with breast cancer. The pooled incidence of atrial fibrillation was 2.7% (95% CI: 1.1 to 4.9%) with high heterogeneity (I^2^ = 99.90%) (Figure 2B).

#### Bidirectional association between breast cancer and atrial fibrillation

**Figure 4:**
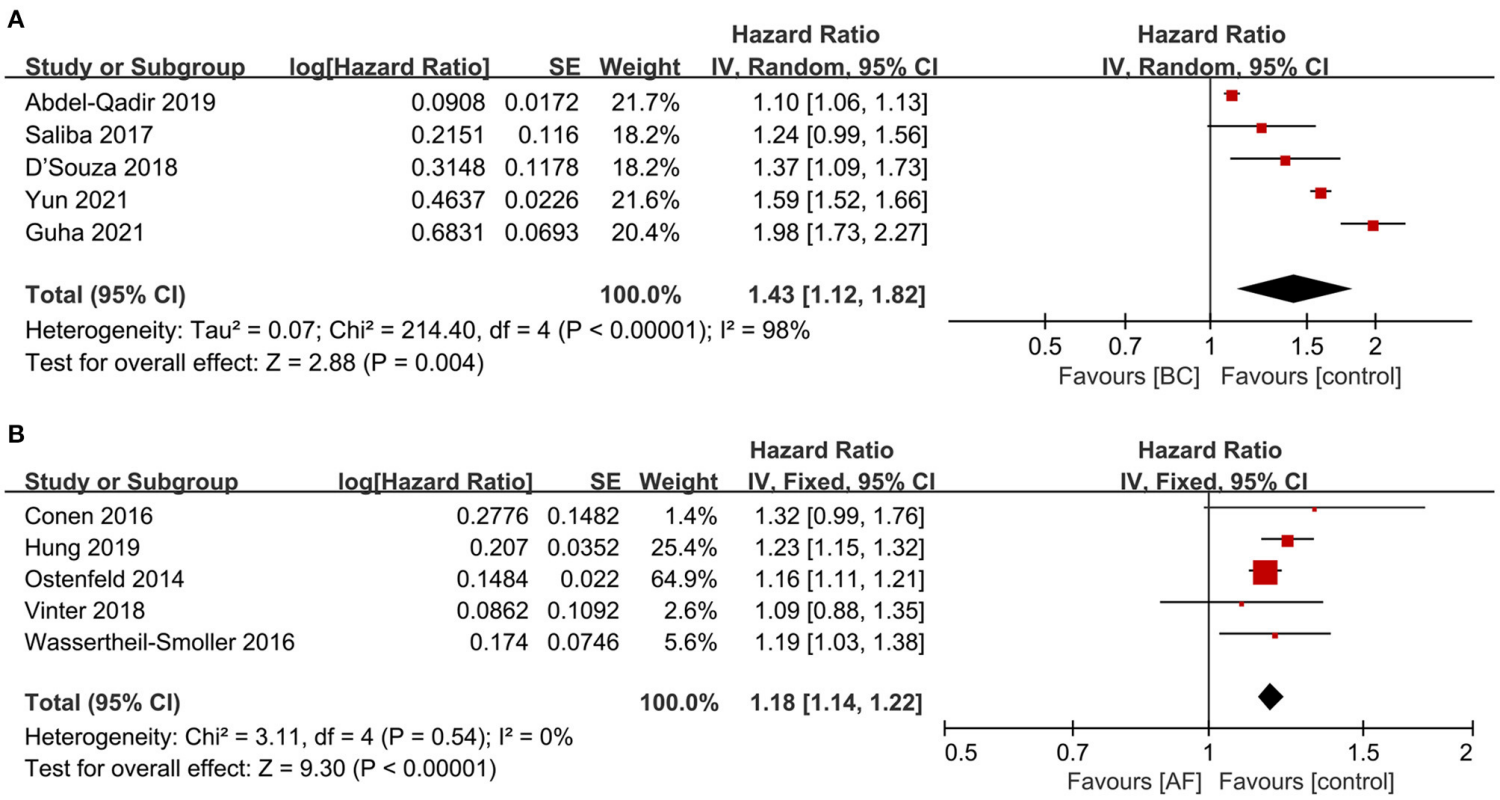
Forest plot of bidirectional association between BC and AF [**(A)** the risk of AF in patients with BC vs. **(B)** the risk of BC in patients with AF]. Inside the forest plot, the rhombus indicates the pooled estimate. Red square indicates study size. Black vertical lines indicate 95%CIs around the effect size estimate. AF, atrial fibrillation; BC, breast cancer.

A total of five articles (7, 10, 11, 13, 14) reported the risk of new-onset atrial fibrillation among breast cancer patients. As shown in Figure 4A, the risk of atrial fibrillation was significantly increased in breast cancer patients compared with non-breast cancer patients in pooled cohort studies (HR: 1.43, 95% CI: 1.12 to 1.82, I^2^ = 98%).

Five articles (16–20) reported the incidence of breast cancer among patients with atrial fibrillation. As shown in Figure 4B, the incidence of breast cancer was significantly increased in patients with atrial fibrillation in pooled cohort studies (HR: 1.18, 95% CI: 1.14 to 1.22, I^2^ = 0%).

According to the predefined criteria, subgroup analysis was not performed due to the limited number of studies.

### Publication bias and sensitivity analysis

As shown in Supplementary Figure S1, funnel plots, Egger's test (both *P* > 0.1), and Begg's test (both *P* > 0.1) showed no significant publication bias in risk of atrial fibrillation, risk of breast cancer and incidence of atrial fibrillation, even though funnel plots are not recommended when the number of articles included is limited (N < 10). However, funnel plots, Egger's (*p* < 0.05), and Begg's tests (*p* < 0.05) for the prevalence of atrial fibrillation suggest publication bias. Sensitivity analysis by omitting each study indicated that our results were stable and reliable, with a range from 1.31 (95% CI: 1.01 to 1.69) to 1.56 (95% CI: 1.32 to 1.83) for the risk of atrial fibrillation and 1.16 (95% CI: 1.12 to 1.21) to 1.21 (95% CI: 1.15 to 1.29) for the risk of breast cancer (Supplementary Figure S2). For the risk of atrial fibrillation, the results remained stable when we used the fixed effects analysis model (HR: 1.28, 95% CI: 1.25 to 1.31) (Supplementary Figure S3).

### Quality assessment

The GRADE tool was used to evaluate the quality of evidence of cohort studies. All the included studies were observational cohort studies, so the initial level of evidence was moderate (37). As there was greater heterogeneity in the risk of atrial fibrillation (I^2^ = 98%), downgrades were given. Ultimately, the risk of atrial fibrillation was evaluated as low certainty, and the risk of breast cancer was evaluated as moderate by the GRADE tool (Supplementary Table S6).

## Discussion

### Major findings

Our results with a sample size of 8,537,551 individuals showed that the prevalence of atrial fibrillation among breast cancer patients was 3% and that incidence was 2.7%. Patients with breast cancer had an increased risk of atrial fibrillation of 43%, and patients with atrial fibrillation had an increased risk of breast cancer of 18% in pooled cohorts, which first showed a bidirectional association between atrial fibrillation and breast cancer (Graphical Abstract).

Age is a vital risk factor for both atrial fibrillation and breast cancer, and the prevalence of both increases with aging (4, 48). A national retrospective cohort study showed that breast cancer was associated with an increased risk of atrial fibrillation from 6 months to 3 years of follow-up, both in age < 60 (HR: 1.80, 95% CI: 1.38–2.35) and age >60 groups (HR: 1.14, 95% CI: 1.05 to 1.25) after adjustments (10). However, a large case-control study with a sample size of 122,100 showed a reduced risk of atrial fibrillation in the age < 65 group (OR: 0.70, 95% CI: 0.69 to 0.71) and 65–80 group (OR: 0.91, 95% CI: 0.90 to 0.92) but an increased risk of atrial fibrillation in the age >80 group (OR: 1.01, 95% CI: 1.00 to 1.02) (15). Our results showed that the prevalence of atrial fibrillation among elderly individuals (8.6%) was higher than that in those aged < 65 years (1.9%). Studies that included atrial fibrillation in patients with breast cancer adjusted for age, so our results suggest that the increased risk of atrial fibrillation in breast cancer patients may be independent of age.

In addition, the treatment of breast cancer patients, such as surgery, chemotherapy and radiation, can also contribute to atrial fibrillation (49, 50). The most commonly used chemotherapy drugs for breast cancer, such as alkylates, anthracyclines, paclitaxel and trastuzumab have been found to be cardiotoxic and can induce atrial fibrillation in breast cancer patients (51, 52). Moreover, pain medications used by patients with advanced cancer, including non-steroidal anti-inflammatory drugs and opioids, are associated with an increased risk of atrial fibrillation (53, 54). A case-control study involving patients first diagnosed with breast cancer who had not been treated showed a significantly increased risk of atrial fibrillation in these patients compared with non-breast cancer patients (12). To some extent, the increased risk of atrial fibrillation in breast cancer patients may be independent of cancer treatment.

Similarly, drug treatment for patients with atrial fibrillation may also play a role in promoting breast cancer, such as amiodarone, which may be associated with an increased risk of cancer (55). In addition, a retrospective cohort study of 2,116,029 women showed a significantly increased risk of breast cancer among patients currently on digoxin compared with a control group (RR: 1.39, 95% CI: 1.32 to 1.46) (56). It is speculated that this may be related to the estrogen-like effect of digoxin. However, the relationship between cardiac glycosides and breast cancer remains unclear. Recent studies have shown that cardiac glycosides exert antitumor effects through different mechanisms (57, 58). A recent study in the Danish Breast Cancer Cohort confirmed that among 49,312 patients enrolled from 1995 to 2008, the overall risk of breast cancer recurrence did not change significantly in the digoxin group compared with the non-digoxin group (HR: 1.13, 95% CI: 0.88 to 1.46) (59). Although antiarrhythmic drugs may contribute to atrial fibrillation due to estrogenic effects, the risk of breast cancer in patients with atrial fibrillation remains significantly higher after adjusting for hormone replacement therapy (20). Furthermore, our subgroup analysis also showed that the association between atrial fibrillation and breast cancer persists regardless of hormone therapy adjustment (Supplementary Figure S3).

Two articles (1 retrospective cohort study and 1 case-control study) (11, 41) reported the association between atrial fibrillation and all-cause mortality in patients with breast cancer. One retrospective cohort study of 85,423 breast cancer patients showed that new-onset atrial fibrillation was associated with increased all-cause mortality in patients with breast cancer after 1 year of follow-up (HR: 2.15, 95% CI: 1.32 to 3.48) (11). In addition, one case-control study of 2,478,598 patients with metastatic cancer showed that atrial fibrillation was associated with increased all-cause mortality in patients with breast cancer (OR: 1.43, 95% CI: 1.15 to 1.78) (41). Previous study showed an increase in all-cause mortality in patients with atrial fibrillation (11, 41). Guha et al. showed that this increased all-cause mortality was mainly due to increased cardiovascular mortality (11). The most common cause of cardiovascular death is heart failure, followed by embolism and stroke. It is noteworthy that the use of anticoagulants reduced all-cause mortality, either because of fewer embolic events or because anticoagulants hindered breast cancer development (60, 61).

### Underlying mechanism

Our study showed a 43% increased risk of atrial fibrillation in patients with breast cancer compared to those without breast cancer. There is accumulating evidence that breast cancer is closely related to the incidence of atrial fibrillation. Several mechanisms have been proposed to explain this association. First, previous studies have confirmed that the increased risk of atrial fibrillation in cancer patients may be related to the systemic inflammatory state caused by cancer. Inflammation has been considered a vital mechanism involved in the initiation and maintenance of atrial fibrillation (62). Inflammatory markers are significantly increased in patients with breast cancer, such as C-reactive protein, tumor necrosis factor-α, interleukin-2, interleukin-6, and interleukin-8 (63). In addition, breast cancer and atrial fibrillation develop through the same inflammatory pathways, such as the NOD-like receptor protein 3 (NLRP-3) inflammasome (62, 64, 65). Second, some studies have also suggested that autonomic dysfunction in cancer patients may contribute to an increased risk of atrial fibrillation. Patients with breast cancer may face long-term physical pain and mental stress, which may increase sympathetic nerve activity and lead to atrial fibrillation (66, 67). However, there is reason to suspect that the medical exposure of cancer patients is far greater than that of non-cancer patients, which may lead to detection bias. However, in a recent study (7), after adjusting for medical exposure, patients with cancer still had a significantly higher risk of atrial fibrillation than those without cancer, suggesting that this detection bias does not hold.

Our study showed an 18% increased risk of breast cancer in patients with atrial fibrillation. The current study can only show the association between breast cancer and atrial fibrillation but cannot prove a causal relationship. It is speculated that the bidirectional relationship between the two diseases may be related to common risk factors, especially the procoagulant state, as thrombin is a drivers of both atrial fibrillation and breast cancer (68). In addition, apoptosis occurs in the development of atrial fibrillation (69), which may lead to the imbalance between proapoptotic and anti-apoptotic factors, thus reducing the apoptosis of cancer cells and promoting the germinal development of cancer (70). However, several studies have shown a significant increase in the risk of cancer after 90 days of atrial fibrillation and a significant decrease after 90 days, although still associated with cancer, suggesting that in addition to the detection bias caused by the medical exposure of atrial fibrillation patients, many atrial fibrillation patients may already have cancer at the time of diagnosis (16, 18, 19). In other words, common risk factors for both breast cancer and atrial fibrillation only may be early signs of occult cancer (16, 18, 19). Finally, although most studies have adjusted for confounding factors, the association may still be the result of residual confounding. Therefore, the mechanism of the association between atrial fibrillation and breast cancer is very complex, and there is no conclusion yet. Further studies are needed to clarify their association.

### Comparison with previous studies

Our research is somewhat pioneering. Two previous meta-analyses reported an increased risk of cancer diagnosis within 90 days in patients with newly developed atrial fibrillation, but these studies focused on all malignancies rather than specific tumor subtypes (71, 72). A recent meta-analysis reported the prevalence of atrial fibrillation in breast cancer patients across different types of cancer therapy (73). We first assessed the bidirectional association of atrial fibrillation and breast cancer patients.

### Clinical implications

The overall prevalence (3%) of atrial fibrillation among breast cancer patients was similar to that of the general population; however, subgroup analysis showed an increased prevalence of atrial fibrillation among breast cancer patients >65 years of age (8.6%). Given the overall low prevalence of atrial fibrillation among breast cancer patients, screening for atrial fibrillation in breast cancer patients and screening for breast cancer in patients with atrial fibrillation are not currently recommended (4), although the prevalence of atrial fibrillation among breast cancer patients is higher than that among non-breast cancer patients. However, our subgroup analysis showed that the prevalence of atrial fibrillation among patients with breast cancer >65 years of age was four times higher than that among younger breast cancer patients and twice as high as that in the general population >65 years of age (4.4%) (4). Therefore, oncologist must be vigilant about the increased risk of atrial fibrillation in elderly patients with breast cancer.

### Study limitations

Our systematic review and meta-analysis had several limitations. First, all the studies included were observational and could not prove causality. However, a bidirectional relationship between breast cancer and atrial fibrillation was found in cohort rather than case-control studies. Most cohort studies were retrospective cohort studies; therefore, reverse causality may still exist, and more prospective studies are needed. Second, most of the studies we included featured female breast cancer patients, and a considerable number of studies included very few male breast cancer patients. Since we could not contact the researchers to obtain the original data, we included these individual male patients. Third, atrial fibrillation is known to contribute significantly to the incidence of stroke. However, none of the studies we included reported an outcome event for stroke; more studies are needed to clarify the outcomes, especially stroke, in patients with atrial fibrillation and breast cancer.

## Conclusions

Atrial fibrillation is not uncommon in patients with breast cancer, and there is an increased risk of atrial fibrillation in breast cancer patients and vice versa. In addition, breast cancer patients with atrial fibrillation had increased all-cause mortality. GRADE assessment indicated low certainty for the risk of atrial fibrillation and moderate certainty for the risk of breast cancer. More studies are needed to determine whether screening for breast cancer in patients with atrial fibrillation is necessary.

## Data availability statement

The raw data supporting the conclusions of this article will be made available by the authors, without undue reservation.

## Author contributions

RQY contributed to the study concept and design and revised the draft. XDY, QWH, and XL performed the search strategy and contributed to database research, acquisition of data, and statistical analyses. All the authors participated in data analysis, reviewed, and approved the final manuscript.
