# Supplementary material for: Atrial fibrillation and breast cancer—Vicious twins? A systematic review and meta-analysis
Source: Front Cardiovasc Med. 2023 Mar 10;10:1113231. doi: 10.3389/fcvm.2023.1113231 (PMC10036368; doi:10.3389/fcvm.2023.1113231)
Supplement: Supplementary file 1 [file Data_Sheet_1.docx]

**Supplementary Table S1. PRISMA Checklist.**

| **Section/topic** | **#** | **Checklist item** | **Reported on page #** |
| --- | --- | --- | --- |
| **TITLE** | | |  |
| Title | 1 | Identify the report as a systematic review, meta-analysis, or both. | 1 |
| **ABSTRACT** | | |  |
| Structured summary | 2 | Provide a structured summary including, as applicable: background; objectives; data sources; study eligibility criteria, participants, and interventions; study appraisal and synthesis methods; results; limitations; conclusions and implications of key findings; systematic review registration number. | 3 |
| **INTRODUCTION** | | |  |
| Rationale | 3 | Describe the rationale for the review in the context of what is already known. | 5 |
| Objectives | 4 | Provide an explicit statement of questions being addressed with reference to participants, interventions, comparisons, outcomes, and study design (PICOS). | 5 |
| **METHODS** | | |  |
| Protocol and registration | 5 | Indicate if a review protocol exists, if and where it can be accessed (e.g., Web address), and, if available, provide registration information including registration number. | 6 |
| Eligibility criteria | 6 | Specify study characteristics (e.g., PICOS, length of follow-up) and report characteristics (e.g., years considered, language, publication status) used as criteria for eligibility, giving rationale. | 6-7 |
| Information sources | 7 | Describe all information sources (e.g., databases with dates of coverage, contact with study authors to identify additional studies) in the search and date last searched. | 6 |
| Search | 8 | Present full electronic search strategy for at least one database, including any limits used, such that it could be repeated. | 6 |
| Study selection | 9 | State the process for selecting studies (i.e., screening, eligibility, included in systematic review, and, if applicable, included in the meta-analysis). | 9-10 |
| Data collection process | 10 | Describe method of data extraction from reports (e.g., piloted forms, independently, in duplicate) and any processes for obtaining and confirming data from investigators. | 7-8 |
| Data items | 11 | List and define all variables for which data were sought (e.g., PICOS, funding sources) and any assumptions and simplifications made. | 7 |
| Risk of bias in individual studies | 12 | Describe methods used for assessing risk of bias of individual studies (including specification of whether this was done at the study or outcome level), and how this information is to be used in any data synthesis. | 8 |
| Summary measures | 13 | State the principal summary measures (e.g., risk ratio, difference in means). | 8 |
| Synthesis of results | 14 | Describe the methods of handling data and combining results of studies, if done, including measures of consistency (e.g., I^2^) for each meta-analysis. | 8 |
| Risk of bias across studies | 15 | Specify any assessment of risk of bias that may affect the cumulative evidence (e.g., publication bias, selective reporting within studies). | 8 |
| Additional analyses | 16 | Describe methods of additional analyses (e.g., sensitivity or subgroup analyses, meta-regression), if done, indicating which were pre-specified. | 9 |
| **RESULTS** | | |  |
| Study selection | 17 | Give numbers of studies screened, assessed for eligibility, and included in the review, with reasons for exclusions at each stage, ideally with a flow diagram. | 9 |
| Study characteristics | 18 | For each study, present characteristics for which data were extracted (e.g., study size, PICOS, follow-up period) and provide the citations. | 10 |
| Risk of bias within studies | 19 | Present data on risk of bias of each study and, if available, any outcome level assessment (see item 12). | 7 |
| Results of individual studies | 20 | For all outcomes considered (benefits or harms), present, for each study: (a) simple summary data for each intervention group (b) effect estimates and confidence intervals, ideally with a forest plot. | 10 |
| Synthesis of results | 21 | Present results of each meta-analysis done, including confidence intervals and measures of consistency. | 11-13 |
| Risk of bias across studies | 22 | Present results of any assessment of risk of bias across studies (see Item 15). | 11 |
| Additional analysis | 23 | Give results of additional analyses, if done (e.g., sensitivity or subgroup analyses, meta-regression [see Item 16]). | 13 |
| **DISCUSSION** | | |  |
| Summary of evidence | 24 | Summarize the main findings including the strength of evidence for each main outcome; consider their relevance to key groups (e.g., healthcare providers, users, and policy makers). | 14-19 |
| Limitations | 25 | Discuss limitations at study and outcome level (e.g., risk of bias), and at review-level (e.g., incomplete retrieval of identified research, reporting bias). | 19 |
| Conclusions | 26 | Provide a general interpretation of the results in the context of other evidence, and implications for future research. | 20 |
| **FUNDING** | | |  |
| Funding | 27 | Describe sources of funding for the systematic review and other support (e.g., supply of data); role of funders for the systematic review. | 20 |

**Supplementary Table S2. Detailed description of the search strategy**

**PubMed**

| **Search number** | **Query** |
| --- | --- |
| 1 | "Atrial Fibrillation"[Mesh] |
| 2 | ((((((((((((((((((((((((Atrial Fibrillations) OR (Fibrillation, Atrial)) OR (Fibrillations, Atrial)) OR (Auricular Fibrillation)) OR (Auricular Fibrillations)) OR (Fibrillation, Auricular)) OR (Fibrillations, Auricular)) OR (Persistent Atrial Fibrillation)) OR (Atrial Fibrillation, Persistent)) OR (Atrial Fibrillations, Persistent)) OR (Fibrillation, Persistent Atrial)) OR (Fibrillations, Persistent Atrial)) OR (Persistent Atrial Fibrillations)) OR (Familial Atrial Fibrillation)) OR (Atrial Fibrillation, Familial)) OR (Atrial Fibrillations, Familial)) OR (Familial Atrial Fibrillations)) OR (Fibrillation, Familial Atrial)) OR (Fibrillations, Familial Atrial)) OR (Paroxysmal Atrial Fibrillation)) OR (Atrial Fibrillation, Paroxysmal)) OR (Atrial Fibrillations, Paroxysmal)) OR (Fibrillation, Paroxysmal Atrial)) OR (Fibrillations, Paroxysmal Atrial)) OR (Paroxysmal Atrial Fibrillations) |
| 3 | ("Atrial Fibrillation"[Mesh]) OR (((((((((((((((((((((((((Atrial Fibrillations) OR (Fibrillation, Atrial)) OR (Fibrillations, Atrial)) OR (Auricular Fibrillation)) OR (Auricular Fibrillations)) OR (Fibrillation, Auricular)) OR (Fibrillations, Auricular)) OR (Persistent Atrial Fibrillation)) OR (Atrial Fibrillation, Persistent)) OR (Atrial Fibrillations, Persistent)) OR (Fibrillation, Persistent Atrial)) OR (Fibrillations, Persistent Atrial)) OR (Persistent Atrial Fibrillations)) OR (Familial Atrial Fibrillation)) OR (Atrial Fibrillation, Familial)) OR (Atrial Fibrillations, Familial)) OR (Familial Atrial Fibrillations)) OR (Fibrillation, Familial Atrial)) OR (Fibrillations, Familial Atrial)) OR (Paroxysmal Atrial Fibrillation)) OR (Atrial Fibrillation, Paroxysmal)) OR (Atrial Fibrillations, Paroxysmal)) OR (Fibrillation, Paroxysmal Atrial)) OR (Fibrillations, Paroxysmal Atrial)) OR (Paroxysmal Atrial Fibrillations)) |
| 4 | "Breast Neoplasms"[Mesh] |
| 5 | ((((((((((((((((((((((((((((((((((((Breast Neoplasm) OR (Neoplasm, Breast)) OR (Breast Tumors)) OR (Breast Tumor)) OR (Tumor, Breast)) OR (Tumors, Breast)) OR (Neoplasms, Breast)) OR (Breast Cancer)) OR (Cancer, Breast)) OR (Mammary Cancer)) OR (Cancer, Mammary)) OR (Cancers, Mammary)) OR (Mammary Cancers)) OR (Malignant Neoplasm of Breast)) OR (Breast Malignant Neoplasm)) OR (Breast Malignant Neoplasms)) OR (Malignant Tumor of Breast)) OR (Breast Malignant Tumor)) OR (Breast Malignant Tumors)) OR (Cancer of Breast)) OR (Cancer of the Breast)) OR (Mammary Carcinoma, Human)) OR (Carcinoma, Human Mammary)) OR (Carcinomas, Human Mammary)) OR (Human Mammary Carcinomas)) OR (Mammary Carcinomas, Human)) OR (Human Mammary Carcinoma)) OR (Mammary Neoplasms, Human)) OR (Human Mammary Neoplasm)) OR (Human Mammary Neoplasms)) OR (Neoplasm, Human Mammary)) OR (Neoplasms, Human Mammary)) OR (Mammary Neoplasm, Human)) OR (Breast Carcinoma)) OR (Breast Carcinomas)) OR (Carcinoma, Breast)) OR (Carcinomas, Breast) |
| 6 | (((((((((((((((((((((((((((((((((((((Breast Neoplasm) OR (Neoplasm, Breast)) OR (Breast Tumors)) OR (Breast Tumor)) OR (Tumor, Breast)) OR (Tumors, Breast)) OR (Neoplasms, Breast)) OR (Breast Cancer)) OR (Cancer, Breast)) OR (Mammary Cancer)) OR (Cancer, Mammary)) OR (Cancers, Mammary)) OR (Mammary Cancers)) OR (Malignant Neoplasm of Breast)) OR (Breast Malignant Neoplasm)) OR (Breast Malignant Neoplasms)) OR (Malignant Tumor of Breast)) OR (Breast Malignant Tumor)) OR (Breast Malignant Tumors)) OR (Cancer of Breast)) OR (Cancer of the Breast)) OR (Mammary Carcinoma, Human)) OR (Carcinoma, Human Mammary)) OR (Carcinomas, Human Mammary)) OR (Human Mammary Carcinomas)) OR (Mammary Carcinomas, Human)) OR (Human Mammary Carcinoma)) OR (Mammary Neoplasms, Human)) OR (Human Mammary Neoplasm)) OR (Human Mammary Neoplasms)) OR (Neoplasm, Human Mammary)) OR (Neoplasms, Human Mammary)) OR (Mammary Neoplasm, Human)) OR (Breast Carcinoma)) OR (Breast Carcinomas)) OR (Carcinoma, Breast)) OR (Carcinomas, Breast)) OR ("Breast Neoplasms"[Mesh]) |
| 7 | ((((((((((((((((((((((((((((((((((((((Breast Neoplasm) OR (Neoplasm, Breast)) OR (Breast Tumors)) OR (Breast Tumor)) OR (Tumor, Breast)) OR (Tumors, Breast)) OR (Neoplasms, Breast)) OR (Breast Cancer)) OR (Cancer, Breast)) OR (Mammary Cancer)) OR (Cancer, Mammary)) OR (Cancers, Mammary)) OR (Mammary Cancers)) OR (Malignant Neoplasm of Breast)) OR (Breast Malignant Neoplasm)) OR (Breast Malignant Neoplasms)) OR (Malignant Tumor of Breast)) OR (Breast Malignant Tumor)) OR (Breast Malignant Tumors)) OR (Cancer of Breast)) OR (Cancer of the Breast)) OR (Mammary Carcinoma, Human)) OR (Carcinoma, Human Mammary)) OR (Carcinomas, Human Mammary)) OR (Human Mammary Carcinomas)) OR (Mammary Carcinomas, Human)) OR (Human Mammary Carcinoma)) OR (Mammary Neoplasms, Human)) OR (Human Mammary Neoplasm)) OR (Human Mammary Neoplasms)) OR (Neoplasm, Human Mammary)) OR (Neoplasms, Human Mammary)) OR (Mammary Neoplasm, Human)) OR (Breast Carcinoma)) OR (Breast Carcinomas)) OR (Carcinoma, Breast)) OR (Carcinomas, Breast)) OR ("Breast Neoplasms"[Mesh])) AND (("Atrial Fibrillation"[Mesh]) OR (((((((((((((((((((((((((Atrial Fibrillations) OR (Fibrillation, Atrial)) OR (Fibrillations, Atrial)) OR (Auricular Fibrillation)) OR (Auricular Fibrillations)) OR (Fibrillation, Auricular)) OR (Fibrillations, Auricular)) OR (Persistent Atrial Fibrillation)) OR (Atrial Fibrillation, Persistent)) OR (Atrial Fibrillations, Persistent)) OR (Fibrillation, Persistent Atrial)) OR (Fibrillations, Persistent Atrial)) OR (Persistent Atrial Fibrillations)) OR (Familial Atrial Fibrillation)) OR (Atrial Fibrillation, Familial)) OR (Atrial Fibrillations, Familial)) OR (Familial Atrial Fibrillations)) OR (Fibrillation, Familial Atrial)) OR (Fibrillations, Familial Atrial)) OR (Paroxysmal Atrial Fibrillation)) OR (Atrial Fibrillation, Paroxysmal)) OR (Atrial Fibrillations, Paroxysmal)) OR (Fibrillation, Paroxysmal Atrial)) OR (Fibrillations, Paroxysmal Atrial)) OR (Paroxysmal Atrial Fibrillations))) |

**Embase**

| **No.** | **Query** |
| --- | --- |
| #1 | 'atrial fibrillation'/exp |
| #2 | 'atrial fibrillations':ab,ti OR 'fibrillation, atrial':ab,ti OR 'fibrillations, atrial':ab,ti OR 'auricular fibrillation':ab,ti OR 'auricular fibrillations':ab,ti OR 'fibrillation, auricular':ab,ti OR 'persistent atrial fibrillation':ab,ti OR 'fibrillations, auricular':ab,ti OR 'atrial fibrillation, persistent':ab,ti OR 'atrial fibrillations, persistent':ab,ti OR 'fibrillation, persistent atrial':ab,ti OR 'fibrillations, persistent atrial':ab,ti OR 'persistent atrial fibrillations':ab,ti OR 'familial atrial fibrillation':ab,ti OR 'atrial fibrillation, familial':ab,ti OR 'atrial fibrillations, familial':ab,ti OR 'familial atrial fibrillations':ab,ti OR 'fibrillation, familial atrial':ab,ti OR 'fibrillations, familial atrial':ab,ti OR 'paroxysmal atrial fibrillation':ab,ti OR 'atrial fibrillation, paroxysmal':ab,ti OR 'atrial fibrillations, paroxysmal':ab,ti OR 'fibrillation, paroxysmal atrial':ab,ti OR 'fibrillations, paroxysmal atrial':ab,ti OR 'paroxysmal atrial fibrillations':ab,ti |
| #3 | #1 OR #2 |
| #4 | 'breast cancer'/exp |
| #5 | 'breast neoplasm':ab,ti OR 'neoplasm, breast':ab,ti OR 'breast tumors':ab,ti OR 'breast tumor':ab,ti OR 'tumor, breast':ab,ti OR 'tumors, breast':ab,ti OR 'neoplasms, breast':ab,ti OR 'breast cancer':ab,ti OR 'mammary cancer':ab,ti OR 'cancer, breast':ab,ti OR 'cancer, mammary':ab,ti OR 'cancers, mammary':ab,ti OR 'mammary cancers':ab,ti OR 'malignant neoplasm of breast':ab,ti OR 'breast malignant neoplasm':ab,ti OR 'breast malignant neoplasms':ab,ti OR 'malignant tumor of breast':ab,ti OR 'breast malignant tumor':ab,ti OR 'breast malignant tumors':ab,ti OR 'cancer of breast':ab,ti OR 'cancer of the breast':ab,ti OR 'mammary carcinoma, human':ab,ti OR 'carcinomas, human mammary':ab,ti OR 'carcinoma, human mammary':ab,ti OR 'human mammary carcinomas':ab,ti OR 'human mammary carcinoma':ab,ti OR 'mammary carcinomas, human':ab,ti OR 'mammary neoplasms, human':ab,ti OR 'human mammary neoplasm':ab,ti OR 'human mammary neoplasms':ab,ti OR 'neoplasm, human mammary':ab,ti OR 'neoplasms, human mammary':ab,ti OR 'mammary neoplasm, human':ab,ti OR 'breast carcinoma':ab,ti OR 'breast carcinomas':ab,ti OR 'carcinoma, breast':ab,ti OR 'carcinomas, breast':ab,ti |
| #6 | #4 OR #5 |
| #7 | #3 AND #6 |

**Cochrane**

| **ID** | **Search** |
| --- | --- |
| #1 | MeSH descriptor: [Breast Neoplasms] explode all trees |
| #2 | (Breast Neoplasm):ti,ab,kw or (Neoplasm, Breast):ti,ab,kw or ( Breast Tumors):ti,ab,kw or (Breast Tumor):ti,ab,kw or (Tumor, Breast):ti,ab,kw or (Tumors, Breast):ti,ab,kw or (Neoplasms, Breast):ti,ab,kw or (Breast Cancer):ti,ab,kw or (Cancer, Breast):ti,ab,kw or (Cancer, Mammary):ti,ab,kw or (Mammary Cancer):ti,ab,kw or (Cancers, Mammary):ti,ab,kw or (Mammary Cancers):ti,ab,kw or (Malignant Neoplasm of Breast):ti,ab,kw or (Breast Malignant Neoplasm):ti,ab,kw or (Breast Malignant Neoplasms):ti,ab,kw or (Malignant Tumor of Breast):ti,ab,kw or (Breast Malignant Tumor):ti,ab,kw or (Breast Malignant Tumors):ti,ab,kw or (Cancer of Breast):ti,ab,kw or (Cancer of the Breast):ti,ab,kw or (Mammary Carcinoma, Human):ti,ab,kw or (Carcinoma, Human Mammary):ti,ab,kw or (Carcinomas, Human Mammary):ti,ab,kw or (Mammary Carcinomas, Human):ti,ab,kw or (Human Mammary Carcinomas):ti,ab,kw or (Human Mammary Carcinoma):ti,ab,kw or (Mammary Neoplasms, Human):ti,ab,kw or (Human Mammary Neoplasm):ti,ab,kw or (Human Mammary Neoplasms):ti,ab,kw or (Neoplasm, Human Mammary):ti,ab,kw or (Neoplasms, Human Mammary):ti,ab,kw or (Breast Carcinoma):ti,ab,kw or (Mammary Neoplasm, Human):ti,ab,kw or (Breast Carcinomas):ti,ab,kw or (Carcinoma, Breast):ti,ab,kw or (Carcinomas, Breast):ti,ab,kw |
| #3 | #1 or #2 |
| #4 | MeSH descriptor: [Atrial Fibrillation] explode all trees |
| #5 | (Atrial Fibrillations):ti,ab,kw or (Fibrillation, Atrial):ti,ab,kw or (Fibrillations, Atrial):ti,ab,kw or (Auricular Fibrillation):ti,ab,kw or (Auricular Fibrillations):ti,ab,kw or (Fibrillation, Auricular):ti,ab,kw or (Fibrillations, Auricular):ti,ab,kw or (Persistent Atrial Fibrillation):ti,ab,kw or (Atrial Fibrillation, Persistent):ti,ab,kw or (Atrial Fibrillations, Persistent):ti,ab,kw or (Fibrillation, Persistent Atrial):ti,ab,kw or (Fibrillations, Persistent Atrial):ti,ab,kw or (Persistent Atrial Fibrillations):ti,ab,kw or (Familial Atrial Fibrillation):ti,ab,kw or (Atrial Fibrillation, Familial):ti,ab,kw or (Atrial Fibrillations, Familial):ti,ab,kw or (Familial Atrial Fibrillations):ti,ab,kw or (Fibrillation, Familial Atrial):ti,ab,kw or (Fibrillations, Familial Atrial):ti,ab,kw or (Paroxysmal Atrial Fibrillation):ti,ab,kw or (Atrial Fibrillation, Paroxysmal):ti,ab,kw or (Atrial Fibrillations, Paroxysmal):ti,ab,kw or (Fibrillation, Paroxysmal Atrial):ti,ab,kw or (Fibrillations, Paroxysmal Atrial):ti,ab,kw or (Paroxysmal Atrial Fibrillations):ti,ab,kw |
| #6 | #4 or #5 |
| #7 | #3 and #6 |

**Supplementary Table S3. Studies excluded (n=35) with reasons.**

| **Studies excluded** | **Reasons** |
| --- | --- |
| Arslan 2010 ^1^ | This included population is not breast cancer patient |
| Chen 2019 ^2^ | Unrelated to incidence of atrial fibrillation |
| Chung 2020 ^3^ | Unrelated to incidence of atrial fibrillation |
| D’Souza 2019 ^4^ | Unrelated to incidence of atrial fibrillation |
| Elbadawi 2017 ^5^ | Unrelated to incidence of atrial fibrillation |
| Erichsen 2011 ^6^ | This included population is not breast cancer patient |
| Fender 2020 ^7^ | This is a review |
| Gegechkori 2019 ^8^ | Unrelated to incidence of atrial fibrillation |
| Giustozzi 2020 ^9^ | This included population is not breast cancer patient |
| Grogan 2007 ^10^ | This included population is not breast cancer patient |
| Hussain 2020 ^11^ | This included population is not breast cancer patient |
| Jiang 2021 ^12^ | This included population is not breast cancer patient |
| Karnik 2018 ^13^ | This is a comment |
| Kolodziejczyk 2021 ^14^ | Unrelated to incidence of atrial fibrillation |
| Lahan 2021^15^ | Unrelated to incidence of atrial fibrillation |
| Lardaro 2015 ^16^ | This included population is not breast cancer patient |
| Lateef 2020 ^17^ | This is a meta-analysis |
| Lee 2016 ^18^ | Unrelated to incidence of atrial fibrillation |
| Mao 2018 ^19^ | This is a review |
| Martinello 2018 ^20^ | Unrelated to incidence of atrial fibrillation |
| Menichelli 2021 ^21^ | This is a review |
| Merino 2021 ^22^ | This is a review |
| Mery 2017 ^23^ | This is a review |
| O'Neal 2015 ^24^ | This included population is not breast cancer patient |
| Pacholczak-Madej 2020 ^25^ | Unrelated to incidence of atrial fibrillation |
| Pardo Sanz 2019 ^26^ | Unrelated to incidence of atrial fibrillation |
| Pastori 2021 ^27^ | This included population is not breast cancer patient |
| Rydzek 2015 ^28^ | This included population is not breast cancer patient |
| Tajiri 2020 ^29^ | This included population is not breast cancer patient |
| Vedovati 2018 ^30^ | Unrelated to incidence of atrial fibrillation |
| Wilkinson 2010 ^31^ | This included population is not breast cancer patient |
| Yang 2018 ^32^ | This is a review |
| Yang 2019 ^33^ | Unrelated to incidence of atrial fibrillation |
| Yuan 2019 ^34^ | This is a meta-analysis |
| Zhang 2020 ^35^ | This is a meta-analysis |

1. Arslan C, Aksoy S, Dizdar O, Dede DS, Harputluoglu H, Altundag K. Zoledronic acid and atrial fibrillation in cancer patients. *Support Care Cancer.* 2011;19(3):425-430.

2. Chen ST, Hellkamp AS, Becker RC, et al. Efficacy and safety of rivaroxaban vs. warfarin in patients with non-valvular atrial fibrillation and a history of cancer: observations from ROCKET AF. *Eur Heart J Qual Care Clin Outcomes.* 2019;5(2):145-152.

3. Chung SY, Oh J, Chang JS, et al. Risk of Cardiac Disease in Patients With Breast Cancer: Impact of Patient-Specific Factors and Individual Heart Dose From Three-Dimensional Radiation Therapy Planning. *Int J Radiat Oncol Biol Phys.* 2021;110(2):473-481.

4. D'Souza M, Smedegaard L, Madelaire C, et al. Atrial fibrillation and anticoagulation in patients with breast cancer. *Scand Cardiovasc J.* 2019;53(5):247-254.

5. Elbadawi A, Elgendy IY, Ha LD, et al. In-Hospital Cerebrovascular Outcomes of Patients With Atrial Fibrillation and Cancer (from the National Inpatient Sample Database). *Am J Cardiol.* 2018;121(5):590-595.

6. Erichsen R, Christiansen CF, Froslev T, Jacobsen J, Sorensen HT. Intravenous bisphosphonate therapy and atrial fibrillation/flutter risk in cancer patients: a nationwide cohort study. *Br J Cancer.* 2011;105(7):881-883.

7. Fender AC, Dobrev D. The anticoagulation dilemma and future treatment avenues in patients with breast cancer and atrial fibrillation. *Int J Cardiol.* 2021;323:194-196.

8. Gegechkori N, Egorova N, Mhango G, Wisnivesky JP, Lin JJ. Bisphosphonate use and incident cardiovascular events among older breast cancer survivors. *The Breast.* 2019;47:28-32.

9. Giustozzi M, Ali H, Reboldi G, et al. Safety of catheter ablation of atrial fibrillation in cancer survivors. *J Interv Card Electrophysiol.* 2021;60(3):419-426.

10. Grogan KM, Wong C, Nutescu EA, Shord SS. Examining differences in weekly warfarin dose in patients with and without cancer. *Therapeutic drug monitoring.* 2007;29(5):638-643.

11. Hussain M, Misbah R, Donnellan E, et al. Impact of timing of atrial fibrillation, CHA2DS2-VASc score and cancer therapeutics on mortality in oncology patients. *Open Heart.* 2020;7(2).

12. Jiang J, Shang X, Zhao J, et al. Score for Predicting Active Cancer in Patients with Ischemic Stroke: A Retrospective Study. *Biomed Res Int.* 2021;2021:5585206.

13. Karnik AA, Benjamin EJ, Trinquart L. Breast cancer and atrial fibrillation-A malignant combination? *Heart Rhythm.* 2019;16(3):349-350.

14. Kolodziejczyk C, Jakobsen M, Sall Jensen M, et al. Mortality from cardiovascular disease in women with breast cancer - a nationwide registry study. *Acta Oncol.* 2021;60(10):1257-1263.

15. Lahan S, Bharadwaj A, Cheng R, et al. In-Hospital Characteristics and 30-Day Readmissions for Acute Myocardial Infarction and Major Bleeding in Patients With Active Cancer. *Am J Cardiol.* 2022;166:25-37.

16. Lardaro T, Self WH, Barrett TW. Thirty-day mortality in ED patients with new onset atrial fibrillation and actively treated cancer. *Am J Emerg Med.* 2015;33(10):1483-1488.

17. Lateef N, Kapoor V, Ahsan MJ, et al. Atrial fibrillation and cancer; understanding the mysterious relationship through a systematic review. *J Community Hosp Intern Med Perspect.* 2020;10(2):127-132.

18. Lee CW, Muo CH, Liang JA, Lin MC, Kao CH. Atrial Fibrillation is Associated With Morphine Treatment in Female Breast Cancer Patients: A Retrospective Population-Based Time-Dependent Cohort Study. *Medicine (Baltimore).* 2016;95(11):e3102.

19. Mao L, Huang W, Zou P, Dang X, Zeng X. The unrecognized role of tumor suppressor genes in atrial fibrillation. *Gene.* 2018;642:26-31.

20. Martinello R, Becco P, Vici P, et al. Trastuzumab-related cardiotoxicity in patients with nonlimiting cardiac comorbidity. *Breast J.* 2019;25(3):444-449.

21. Menichelli D, Vicario T, Ameri P, et al. Cancer and atrial fibrillation: Epidemiology, mechanisms, and anticoagulation treatment. *Prog Cardiovasc Dis.* 2021;66:28-36.

22. Merino JL. Atrial fibrillation and breast cancer: casual or causal relationship? *Eur Heart J.* 2022;43(4):313-315.

23. Mery B, Guichard JB, Guy JB, et al. Atrial fibrillation in cancer patients: Hindsight, insight and foresight. *Int J Cardiol.* 2017;240:196-202.

24. O'Neal WT, Lakoski SG, Qureshi W, et al. Relation between cancer and atrial fibrillation (from the REasons for Geographic And Racial Differences in Stroke Study). *Am J Cardiol.* 2015;115(8):1090-1094.

25. Pacholczak-Madej R, Bazan-Socha S, Zareba L, Undas A, Dropinski J. Direct oral anticoagulants in the prevention of stroke in breast cancer patients with atrial fibrillation during adjuvant endocrine therapy: A cohort study. *Int J Cardiol.* 2021;324:78-83.

26. Pardo Sanz A, Rincon LM, Guedes Ramallo P, et al. Current status of anticoagulation in patients with breast cancer and atrial fibrillation. *Breast.* 2019;46:163-169.

27. Pastori D, Marang A, Bisson A, et al. Thromboembolism, mortality, and bleeding in 2,435,541 atrial fibrillation patients with and without cancer: A nationwide cohort study. *Cancer.* 2021;127(12):2122-2129.

28. Rydzek J, Gasior ZT, Dabek J, Wojnar J, Skrzypek M. Assessment of risk factors for mortality in patients with cardiovascular disease and a history of treatment for malignancy. *Kardiol Pol.* 2015;73(9):730-739.

29. Tajiri K, Sekine I, Naito H, et al. Cardiology consultation in oncology practice: a 5-year survey. *Jpn J Clin Oncol.* 2020;50(12):1419-1425.

30. Vedovati MC, Giustozzi M, Verdecchia P, et al. Patients with cancer and atrial fibrillation treated with doacs: A prospective cohort study. *Int J Cardiol.* 2018;269:152-157.

31. Wilkinson GS, Baillargeon J, Kuo YF, Freeman JL, Goodwin JS. Atrial fibrillation and stroke associated with intravenous bisphosphonate therapy in older patients with cancer. *J Clin Oncol.* 2010;28(33):4898-4905.

32. Yang S, Kwak S, Kwon S, et al. Association of Total Reproductive Years With Incident Atrial Fibrillation, and Subsequent Ischemic Stroke in Women With Natural Menopause. *Circ Arrhythm Electrophysiol.* 2019;12(11):e007428.

33. Yang X, Li X, Yuan M, et al. Anticancer Therapy-Induced Atrial Fibrillation: Electrophysiology and Related Mechanisms. *Front Pharmacol.* 2018;9:1058.

34. Yuan M, Zhang Z, Tse G, et al. Association of Cancer and the Risk of Developing Atrial Fibrillation: A Systematic Review and Meta-Analysis. *Cardiol Res Pract.* 2019;2019:8985273.

35. Zhang M, Li LL, Zhao QQ, et al. The Association of New-Onset Atrial Fibrillation and Risk of Cancer: A Systematic Review and Meta-Analysis. *Cardiol Res Pract.* 2020;2020:2372067.

**Supplementary Table S4. Joanna Briggs Institute critical appraisal checklist applied for included studies.**

| Study | Sample was representative? | Participants appropriately recruited? | Sample size was adequate? | Study subjects and the setting described | Data analysis conducted | Objective, standard criteria, reliably used? | Appropriate statistical analysis used? | Confounding factors/ subgroups/ differences identified and accounted? | Subpopulations identified using objective criteria |
| --- | --- | --- | --- | --- | --- | --- | --- | --- | --- |
| Abdel-Qadir 2019 | Yes | Yes | Yes | Yes | Yes | Yes | Yes | Yes | Yes |
| Abdel-Qadir(2) 2019 | Yes | Yes | Yes | Yes | Yes | Yes | Yes | Yes | Yes |
| Apte 2021 | Yes | Yes | Yes | Yes | Yes | Yes | Yes | Yes | Yes |
| Boerman 2017 | Yes | Yes | Yes | Yes | Yes | Yes | Yes | Yes | Yes |
| D’Souza 2018 | Yes | Yes | Yes | Yes | Yes | Yes | Yes | Yes | Yes |
| Ezaz 2014 | Yes | Yes | Yes | Yes | Yes | Yes | Yes | Yes | Yes |
| Guha 2021 | Yes | Yes | Yes | Yes | Yes | Yes | Yes | Yes | Yes |
| Guzzetti 2008 | Yes | Yes | Yes | Yes | Yes | Yes | Yes | No | Yes |
| Han 2021 | Yes | Yes | Yes | Yes | Yes | Yes | Yes | Yes | Yes |
| Li 2021 | Yes | Yes | Yes | Yes | Yes | Yes | Yes | Yes | Yes |
| Lin 2018 | Yes | Yes | Yes | Yes | Yes | Yes | Yes | Yes | Yes |
| Mery 2020 | Yes | Yes | Yes | Yes | Yes | Yes | Yes | Yes | Yes |
| Okura 2019 | Yes | Yes | Yes | Yes | Yes | Yes | Yes | Yes | Yes |
| Ording 2014 | Yes | Yes | Yes | Yes | Yes | Yes | Yes | Yes | Yes |
| Yamashita 2021 | Yes | Yes | Yes | Yes | Yes | Yes | Yes | Unclear | Unclear |
| Yun 2021 | Yes | Yes | Yes | Yes | Yes | Yes | Yes | Yes | Yes |
| Zubair 2021 | Yes | Yes | Yes | Yes | Yes | Yes | Yes | Yes | Yes |

**Supplementary Table S5. Quality assessment of the included studies by Newcastle–Ottawa scale.**

| Author  (Publication Year) | Newcastle-Ottawa Scale | | | | | | | | | |
| --- | --- | --- | --- | --- | --- | --- | --- | --- | --- | --- |
|  | Selection | | | Comparability | | | Outcome | | | Total |
|  | a | b | c | d | e | f | g | h | i |  |
| Abdel-Qadir 2019, Canada | 1 | 1 | 1 | 1 | 1 | 1 | 1 | 1 | 0 | 8 |
| Conen 2016, USA | 1 | 1 | 1 | 1 | 1 | 1 | 1 | 1 | 0 | 8 |
| D’Souza 2018, Denmark | 1 | 1 | 1 | 1 | 1 | 1 | 1 | 1 | 0 | 8 |
| Guha 2021, USA | 1 | 1 | 1 | 1 | 1 | 1 | 1 | 1 | 1 | 9 |
| Hung 2019, China Taiwan | 1 | 1 | 1 | 1 | 0 | 0 | 1 | 1 | 0 | 6 |
| Ostenfeld 2014, Denmark | 1 | 1 | 1 | 1 | 0 | 0 | 1 | 1 | 0 | 6 |
| Saliba 2017, Israel | 1 | 1 | 1 | 1 | 1 | 1 | 1 | 1 | 0 | 8 |
| Vinter 2018, Denmark | 1 | 1 | 1 | 1 | 1 | 1 | 1 | 1 | 1 | 9 |
| Wassertheil-Smoller 2017, USA | 1 | 1 | 1 | 1 | 1 | 1 | 1 | 1 | 1 | 9 |
| Yun 2021, Korea | 1 | 1 | 1 | 1 | 1 | 1 | 1 | 1 | 0 | 8 |

1. Representativeness of the exposed cohort.
2. Selection of the non-exposed cohort.
3. Ascertainment of exposure.
4. Demonstration that outcome of interest was not present at start of study.
5. Comparability of cohorts on the basis of the design or analysis (adjusted for age).
6. Comparability of cohorts on the basis of the design or analysis (adjusted for any other factor).
7. Assessment of outcome.
8. Was follow-up long enough for outcomes to occur. (1 years for new-onset AF).
9. Adequacy of follow-up of cohorts.

| Author  (Publication Year) | Newcastle-Ottawa Scale | | | | | | | | | |
| --- | --- | --- | --- | --- | --- | --- | --- | --- | --- | --- |
|  | Selection | | | Comparability | | | Outcome | | | Total |
|  | a | b | c | d | e | f | g | h | i |  |
| Apte 2021, USA | 1 | 1 | 0 | 1 | 1 | 1 | 1 | 1 | 0 | 7 |
| Guzzetti 2008, Italy | 1 | 1 | 0 | 1 | 0 | 0 | 1 | 1 | 0 | 5 |
| Zubair 2021, USA | 1 | 1 | 0 | 1 | 1 | 0 | 1 | 1 | 0 | 6 |
| Boerman 2017, Netherland | 1 | 1 | 1 | 1 | 0 | 0 | 1 | 1 | 0 | 6 |

1. Is the case definition adequate.
2. Representativeness of the cases.
3. Selection of Controls.
4. Definition of Controls.
5. Comparability of cases and controls on the basis of the design or analysis (adjusted for age).
6. Comparability of cases and controls on the basis of the design or analysis (adjusted for any other factor).
7. Ascertainment of exposure.
8. Same method of ascertainment for cases and controls.
9. Non-Response rate.

**Supplementary Table S6. GRADE evidence profile**

| **Certainty assessment** | | | | | | | **№ of patients** | | **Effect** | | **Certainty** | **Importance** |
| --- | --- | --- | --- | --- | --- | --- | --- | --- | --- | --- | --- | --- |
| **№ of studies** | **Study design** | **Risk of bias** | **Inconsistency** | **Indirectness** | **Imprecision** | **Other considerations** | **outcome** | **control** | **Relative (95% CI)** | **Absolute (95% CI)** |  |  |
| **Risk of atrial fibrillation (follow-up: range 0.24 years to 10 years; assessed with: ICD10 or ICD9)** | | | | | | | | | | | | |
| 5 | observational studies | not serious | serious^a^ | not serious | not serious | none | 6655/160988 (4.1%) | 17231/445515 (3.9%) | **HR 1.43** (1.12 to 1.82) | **16 more per 1,000** (from 5 more to 31 more) | ⨁⨁◯◯ Low | CRITICAL |
| **Risk of breast cancer (follow-up: range 3.1 years to 19.1 years; assessed with: ICD10 or ICD9)** | | | | | | | | | | | | |
| 5 | observational studies | not serious | not serious | not serious | not serious | none | 3039/418735 (0.7%) | 2582/418735 (0.6%) | **HR 1.18** (1.14 to 1.22) | **1 more per 1,000** (from 1 more to 1 more) | ⨁⨁⨁◯ Moderate | CRITICAL |

a. I^2^=98%

Abbreviation: CI: confidence interval; OR: odds ratio

**Supplementary Figure S1. Funnel plots,** **Egger’s publication bias plot and Begg’s funnel plot.**

a. risk of atrial fibrillation; b. risk of breast cancer; c. prevalence; d. incidence

**
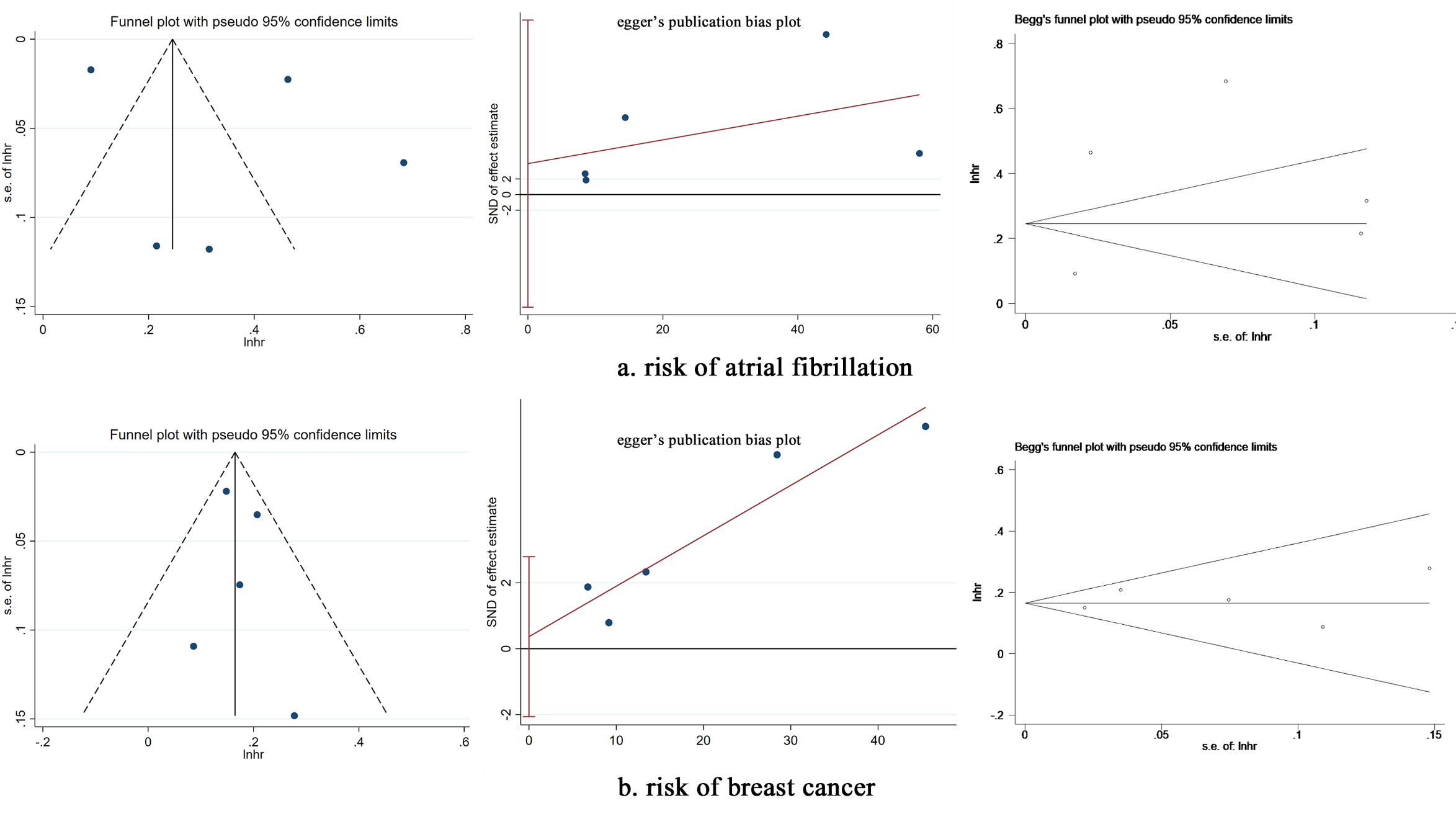
**

**
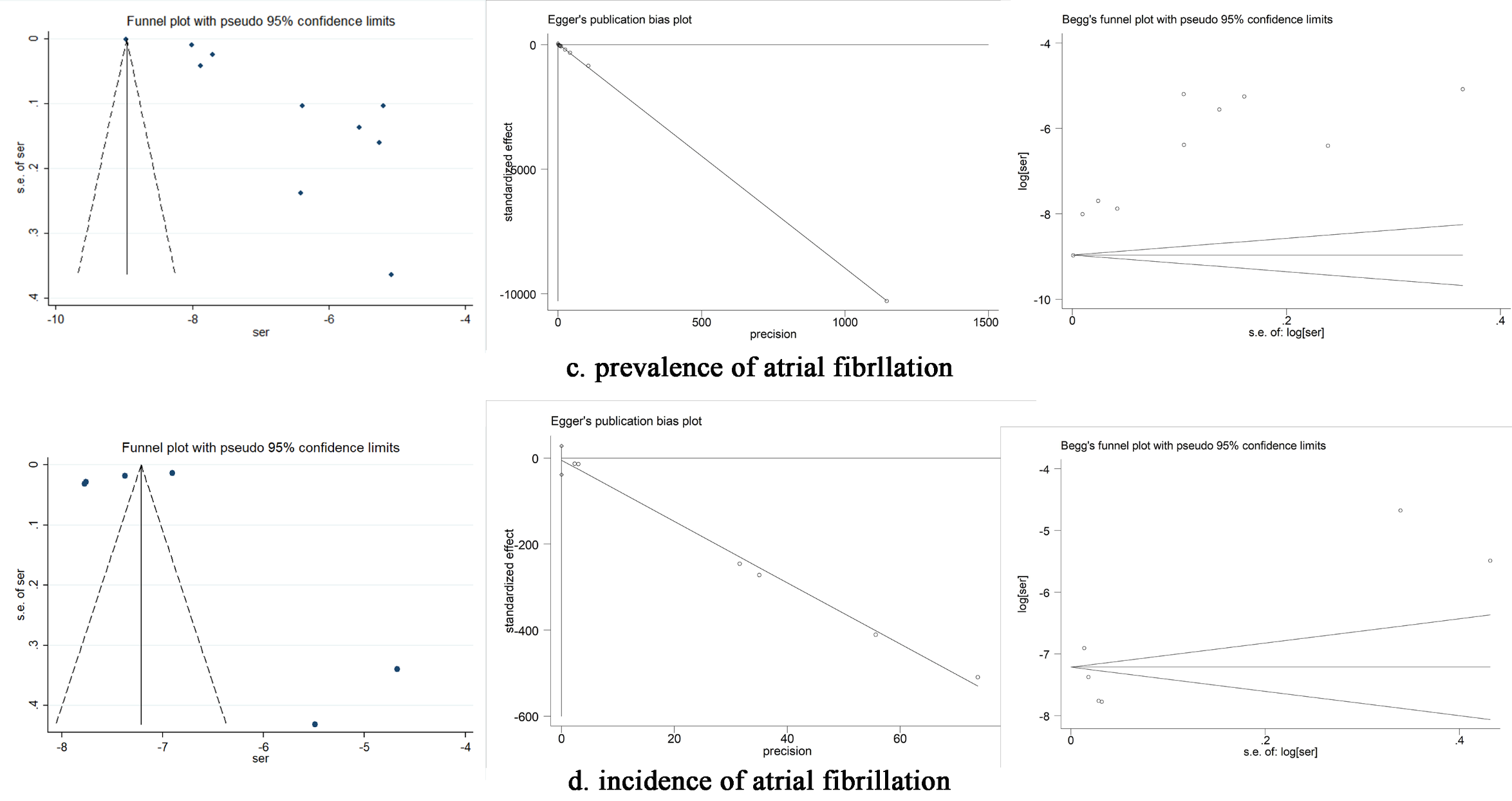
**

**Supplementary Figure S2. Sensitivity analysis.**

a. risk of atrial fibrillation; b. risk of breast cancer**
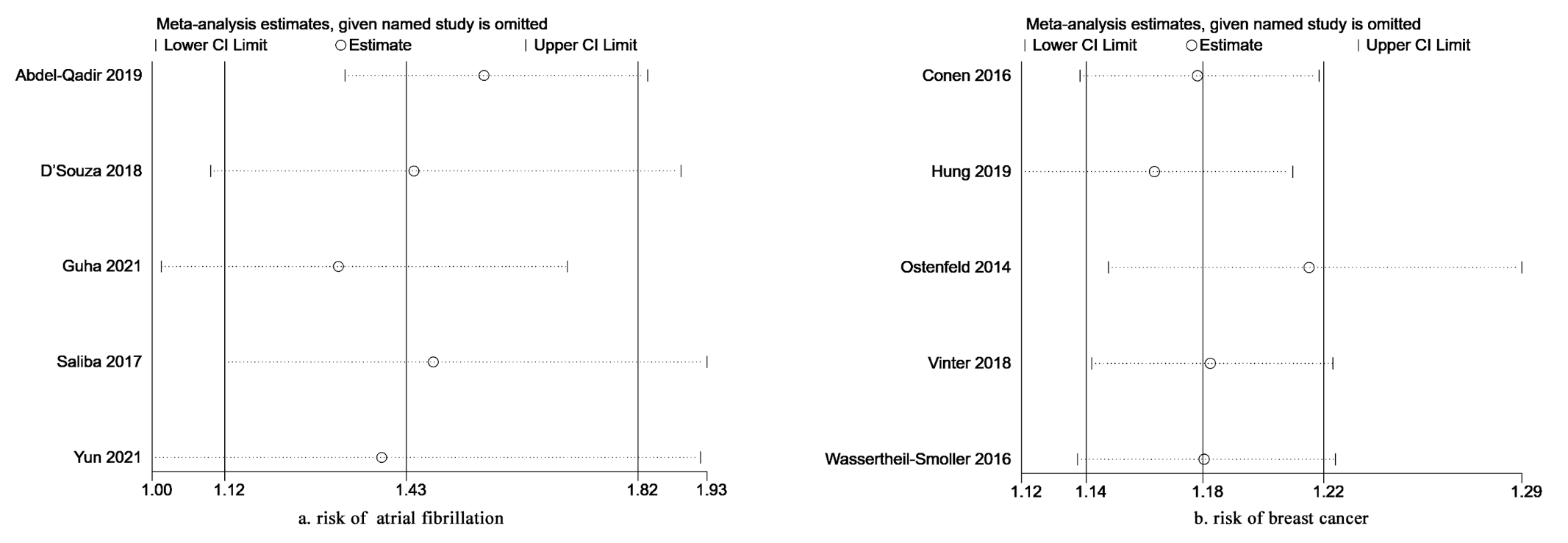
**

**Supplementary Figure S3.** **Fixed-effect analysis model for risk of atrial fibrillation**


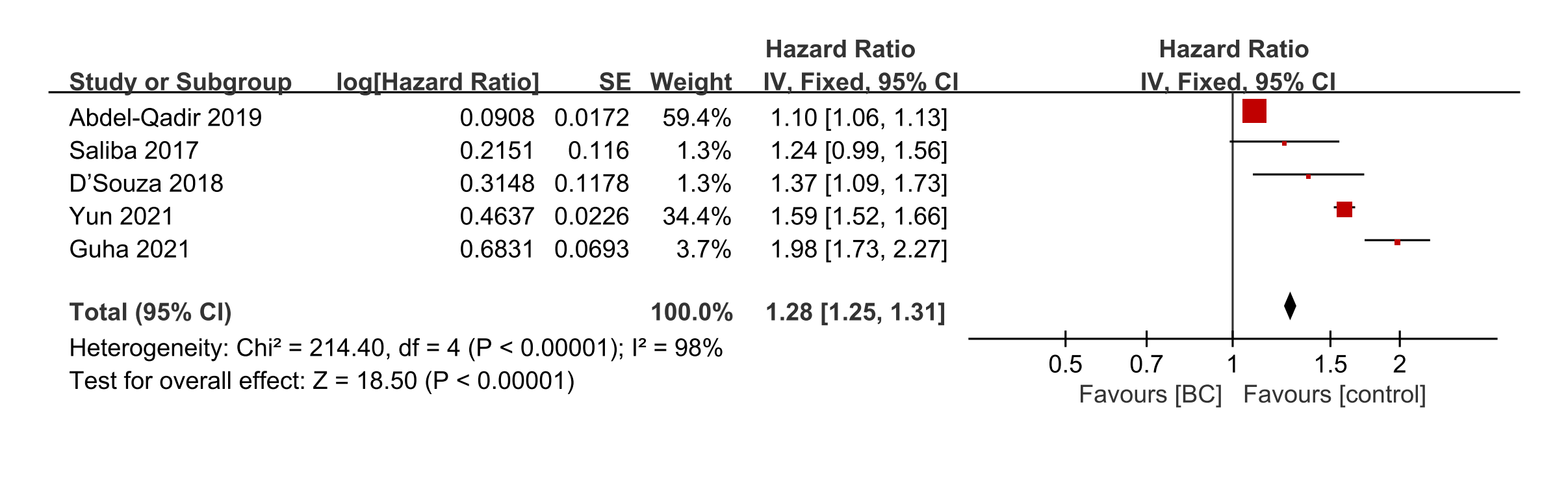


**Supplementary Figure S4. Subgroup analysis for hormone therapy.**


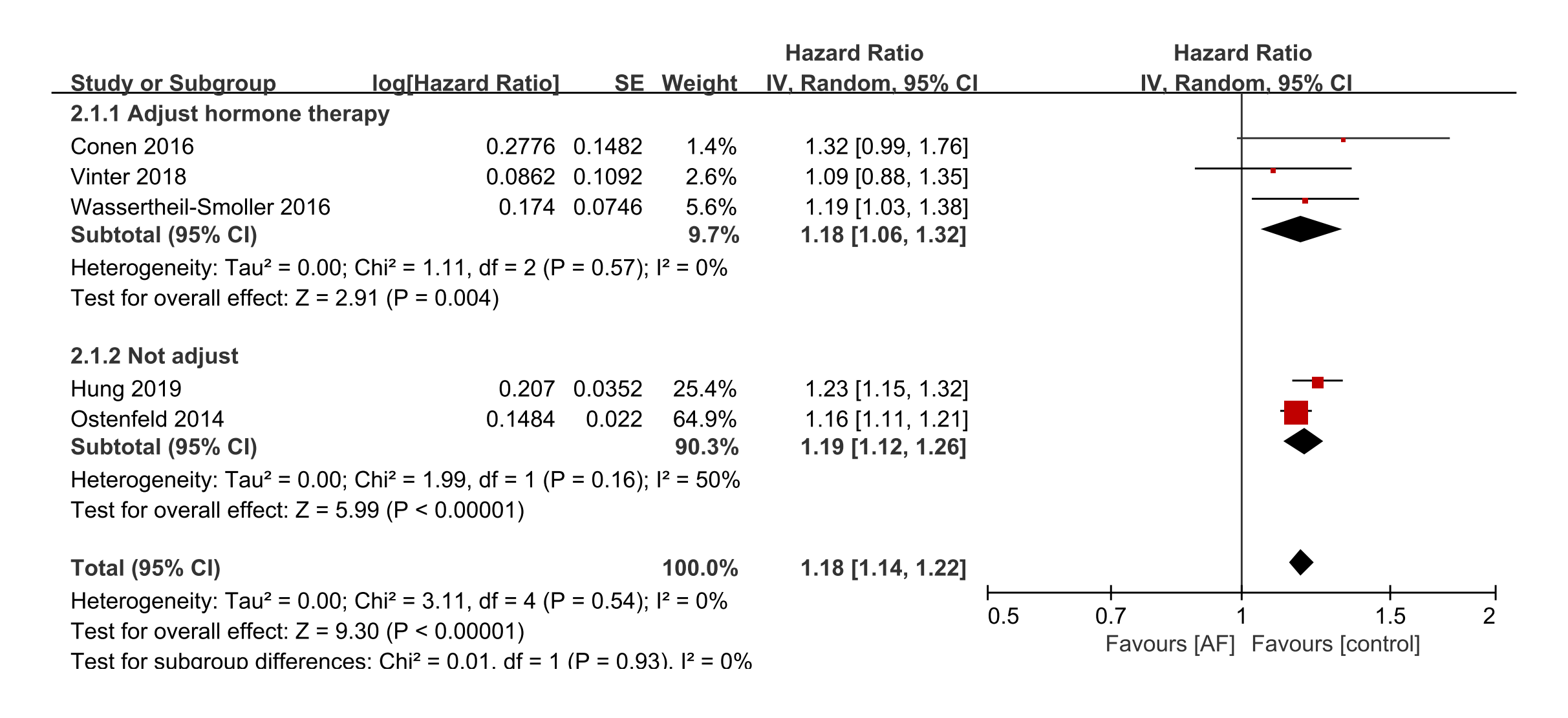


**Supplementary Figure S5. Heterogeneity analysis**

1. **Meta-regression for age; B. Meta-regression for sample size; C. M** **Meta-regression for case; D. subgroup for region**

**
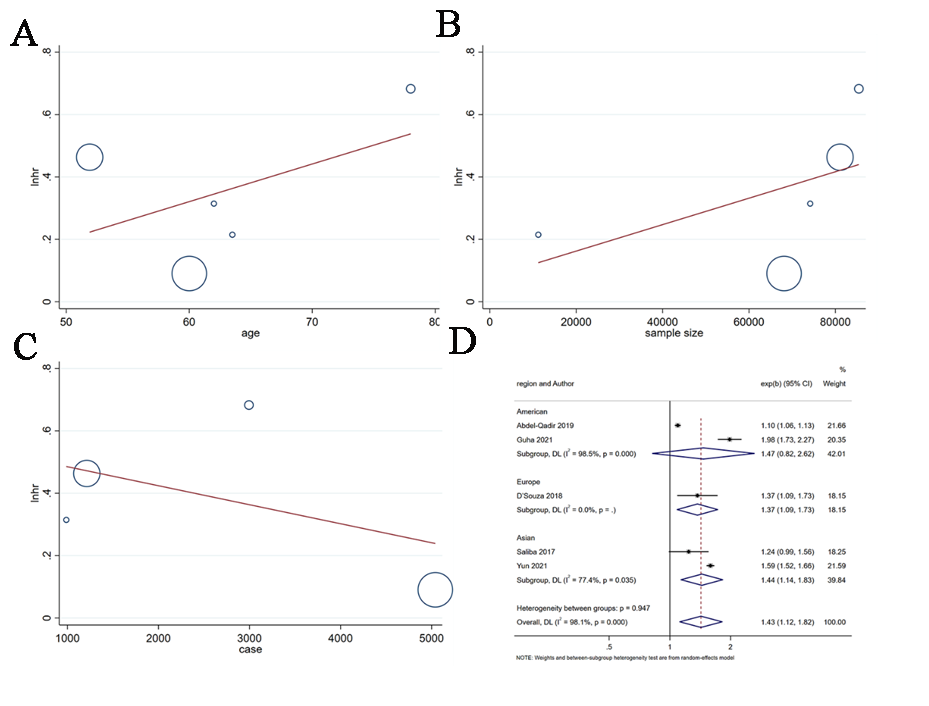
**
